# Artificial Intelligence (AI) and Healthcare Capabilities: A Systematic Literature Review

**DOI:** 10.12688/f1000research.158477.1

**Published:** 2025-01-03

**Authors:** Dereje Ferede

**Affiliations:** 1 Information Systems, Addis Ababa University, Addis Ababa, 1000, Ethiopia

**Keywords:** Artificial Intelligence, Global South, Healthcare Capability, Systematic Literature Review, ICT4D, capability approach, conceptual framework, Digital health, Health information systems, ICT4D, information systems

## Abstract

Artificial Intelligence (AI) has the potential to transform the healthcare ecosystem, but further research is needed to understand how it can enhance healthcare capabilities. This study analyzes the literature on AI and healthcare capability using the PRISMA approach, applying specific search keywords and inclusion/exclusion criteria. The findings indicate that AI benefits the healthcare ecosystem, significantly influences health outcomes, and transforms medical practices. However, there is limited literature and a lack of understanding regarding how AI enhances healthcare capabilities. Most studies date from 2019, suggesting that COVID-19 has accelerated the adoption of AI systems in healthcare. This research contributes theoretically by developing a framework that clarifies AI’s role in enhancing healthcare capabilities, serving as a foundational model for future studies. It identifies critical gaps in the literature, especially in the Global South, and encourages exploration in under-researched areas where healthcare professionals can benefit from AI. Additionally, it bridges the gap between AI and healthcare, enriching interdisciplinary dialogue relevant to emerging economies facing financial constraints. Practically, the study provides actionable insights for healthcare practitioners and policymakers in the Global South on leveraging AI to improve service delivery. It sets the stage for empirical research, promoting the testing and refinement of the proposed framework in resource-limited contexts, while raising awareness among healthcare staff, managers, and technology developers about AI’s role in healthcare.

## Introduction

This systematic literature review analyzes diverse sources to enhance understanding of the nexus between artificial intelligence (AI) and healthcare capabilities, particularly within the framework of ICT4D. AI, a technology designed to replicate human cognitive functions ( Jiang et al., 2017), has the potential to generate significant value across various sectors ( Mikalef et al., 2019). However, its specific contributions to the healthcare ecosystem remain underexplored.

This study highlights a notable gap in AI research, particularly regarding systematic analyses of its relationship with healthcare capabilities. The role of AI-enabled technologies in the healthcare sector is emerging as a crucial area of inquiry ( Zhao, 2018), yet published research remains inadequate ( Mikalef et al., 2019). Moreover, contributions related to business-oriented AI are minimal, indicating a pressing need for studies focused on AI applications in developing markets, especially in light of present and future health crises ( Bharati, 2020). Further research is essential to explore how AI can support sustainable development goals while addressing potential trade-offs ( Gupta et al., 2021). Future studies on AI for sustainability should consider various value perspectives to illustrate how AI can deliver immediate and impactful solutions ( Nishant et al., 2020).

Consequently, this study aims to review the literature and generate insights on effectively applying AI within the healthcare ecosystem. It specifically addresses the research question: How does artificial intelligence empower healthcare capabilities? To answer this question and fulfill the study’s objectives, the article is organized as follows: it begins with a description of the methodology used to identify the nexus between AI and healthcare constructs in the literature, followed by the results. The discussion and conclusion sections will present the constructed conceptual model.

## Methods

This study adopts the PRISMA approach to conduct a systematic literature review on AI and healthcare capability, with a particular emphasis on the implications for ICT4D. By utilizing the PRISMA framework, we aim to enhance the rigor, transparency, and relevance of our findings, ultimately fostering the development and implementation of AI-driven healthcare solutions grounded in solid evidence. This approach is vital in understanding how AI can contribute to healthcare improvements, especially in resource-limited settings typical of many developing regions. The PRISMA methodology has been widely employed in systematic literature reviews within the context of AI and healthcare. For instance, Abdolkhani et al. (2022) utilized this method to investigate the impact of digital health transformation, driven by COVID-19, on nursing practice, highlighting the transformative potential of technology in healthcare. Similarly, Choudhury and Asan (2020) applied this technique to explore the role of artificial intelligence in enhancing patient safety outcomes. By implementing the PRISMA approach, this study aims to systematically review the literature on AI and healthcare capabilities, drawing insights that are particularly relevant to the context of ICT4D. This will help identify pathways through which AI can empower healthcare systems, especially in developing regions, thereby promoting equitable access to advanced healthcare technologies.

### Search strategy

This systematic literature review explores different reference materials from the PubMed database using different searching keywords. Table 1 shows search keywords and sources of literature this systematic literature study used during the examining process.

**Table 1.** Search keywords and literature resources.

Database queried	PubMed
Search keywords	Artificial Intelligence and Healthcare Capability

### Selection criteria

This research performs a scoping (initial) review of nearly a total of 759 studies which are related literature to AI and healthcare capability from PubMed database as per the data available by 22 May 2024. When we limit it to free full text and articles published after 2017, we get a total of 700 papers. This study applies exclusion and inclusion criteria (discussing or reporting the role of AI for healthcare capabilities, published in English language) and came up with 636 irrelevant, 64 not irrelevant articles. Out of the 64 articles, this study drops 9 articles because of being irrelevant, as they are not within the scope of the study. In other words, 64 articles passed to the next screening process. Finally, from 64 articles, this study drops 9 articles due to redundancy. As a result, we perform a systematic review with 46 articles in the domain area of AI and healthcare capabilities (see Table 2).

**Table 2.** Summary of Reviewed Literatures.

No.	Reference	Objective	Methods used	Key findings
1.	( Ahuja, 2019)	• The objective of the study was assessing the impact of AI in medicine to better understand AI enabled technologies and ways of transforming medicine. Furthermore, it examines the AI-enabled technologies' role in performing medical works such as pathology, ophthalmology, radiology, and cardiology.	• Both qualitative and quantitative articles have been reviewed. • Survey methodology applied.	• This paper revealed that AI will enhance physicians and are not likely to substitute the traditional patient- physician association.
2.	( Alhashmi et al., 2019)	• The objective of the study was exploring critical success factors for the adoption of AI in the healthcare sector.	• An extended ETAM model was developed and tested using a qualitative study.	• When deciding whether to use AI in the healthcare industry, managerial, organizational, operational, and IT infrastructure elements should be taken into account as crucial success factors since they have a favourable impact on perceived usefulness and perceived ease of use.
3.	( Asan & Choudhury, 2021)	• The goal of this study was to look into the contributions made by significant human factors communities in applications of artificial intelligence in healthcare. It also examined a number of emerging research gaps and provided future research recommendations.	• The Scopus Master List's "Human Factors and Ergonomics" category undertook an intensive mapping review to collect all pertinent articles published within the last ten years in the major human factors journals and conference sessions.	• The review revealed a nascent but growing body of literature focusing on augmenting health care AI; however, little effort has been made to ensure ecological validity with user-centered design approaches.
4.	( Bohr & Memarzadeh, 2020)	• The objective of the study was to examine the application of AI in the healthcare sector.	• Literature review	• AI has the potential to significantly advance healthcare across the board, from diagnosis to therapy. • Instead of replacing the work of doctors and other healthcare workers as such, technology will facilitate and improve human work. • AI is prepared to assist medical staff with a range of duties, including administrative workflow, clinical documentation, patient outreach, and specialist support like image analysis, medical device automation, and patient monitoring.
5.	( Choudhury & Asan, 2020)	• The objective of the study was identifying and analysing quantitative studies utilizing or integrating AI to address and report clinical-level patient safety outcomes.	• The authors used only the PubMed, PubMed Central, and Web of Science databases to retrieve research articles published in English between January 2009 and August 2019. It focused on quantitative studies.	• The paper revealed that patient safety subcategories, the most frequently used AI, and reported performance metrics. It indicated the lack of a standardized benchmark and heterogeneity in AI reporting. It also showed that AI enabled decision support systems, when implemented correctly, can aid in enhancing patient safety by improving error detection, patient stratification, and drug management.
6.	( D’Antonoli, 2020)	• The objective of the study was to understand the potential risks and hazards that come with AI.	• Literature review	• To reflect time-honoured ethical and legal standards while adequately protecting patient interests, we must apply AI in the best way possible. While providing an overview of the statements that were offered for the ethics of AI applications in radiology, these issues are examined in the context of fundamental biomedical ethics principles and principles for ethical challenges unique to artificial intelligence.
7.	( Davenport & Kalakota, 2019)	• The objective of the study was assessing the potential of AI in health.	• Literature review	• The findings of this study revealed that AI in healthcare can perform diagnosis and treatment recommendations, patient engagement and adherence, and administrative activities.
8.	( Fletcher et al., 2021)	• The objective of the study was to understand the principles AI-enabled systems measured and to illustrate how these principles can be applied to the diagnosis and screening of pulmonary disease.	• Case study	• The three fundamental criteria appropriateness (is the process of choosing how to apply the algorithm in the specific situation and properly adjusting the machine learning model to the intended audience), fairness (entails assessing how different demographic groups are affected and selecting one of several mathematical definitions of group fairness that will effectively fulfil the appropriate set of legal, cultural, and ethical standards), and bias (is a model's systematic propensity to favour one demographic group over another, which can be countered but can result in injustice) can be used to help evaluate the use of AI-enabled systems.
9.	( Floridi et al., 2018)	• The objective of the study was to explore opportunities and risks of AI for society	• Synthesis of literature	• Present a summary of five ethical principles that should guide the development and acceptance of AI, as well as the main opportunities and hazards it poses to society.
10.	( Gama et al., 2022)	• To identify the implementation frameworks used to understand the application of AI in healthcare.	• A scoping review was conducted using the Cochrane.	• Knowledge on how to apply AI in clinical practice is still being developed. • Mention the need for more study and the chance to use already-existing knowledge from the field of implementation science when developing implementation frameworks to direct the future implementation of AI in clinical practice.
11.	( Gerke et al., 2020)	• This paper maps the ethical and legal challenges posed by AI in healthcare and suggests directions for resolving them.	• Literature review	• Informed permission to use, safety and openness, algorithmic fairness and biases, and data privacy are the four main ethical issues. • Safety and efficacy are the first legal challenge, followed by liability, data protection and privacy, cyber security, and intellectual property law.
12.	( Goralski & Tan, 2020)	• Examining the link perspectives of public policy and business strategy to analyse AI impacts on SDGs, and draw practices on leadership development and managerial learning for SDGs were the objective of this study.	• Three case studies have been used in this study.	• This study articulated that AI plays a role in achieving the SDGs. It also draws some lessons on managerial learning and leadership development for global sustainability.
13.	( Gordon, 2021)	• To provide an excellent overview of current debates in the realm of AI and law. • To examine the ethical, legal, and socio-political implications of AI and law.	• Literature review	• The papers show, among other things—perhaps not surprisingly—that the current legal system is unprepared to address the pressing problems brought on by the rapid advancements in AI technology. • To address current and future problems including AI decision-making bias, electronic personhood, and legal liability for autonomous computers, adequate AI regulation is required.
14.	( Guan, 2019)	• Three areas of AI in medicine and healthcare are examined and discussed: the use and potential of AI, particular ethical issues with AI in various frontier disciplines, and potential ethical governance structures.	• Literature review	• The development of an ethical global governance structure and system as well as particular criteria for frontier AI applications in medicine are recommended in order to assure "trustworthy" AI applications in healthcare and medicine.
15.	( Gupta et al., 2021)	• To assess the significant enabling and inhibiting influence of AI for sustainable development.	• A panel discussion with a wide spectrum of AI specialists.	• The significance of AI in accomplishing the Sustainable Development Goals (SDGs); AI for a successful 21st century; Transparency, automated decision-making processes, and personal profiling; and Measuring the relevance of digitalization and artificial intelligence (D&AI) at the indicator level of SDGs. • The need to look beyond the sector-specific silos in which AI is being developed in order to comprehend the potential effects AI may have on societal, environmental, and economic results.
16.	( Gwagwa et al., 2020)	• To provide an overview of the main elements of AI deployment in Africa, Al's core benefits and challenges in African settings, and Al's core policy dimensions for the continent.	• Literature review	• Policymakers are said to need to be aware of the following crucial aspects: gender equity, cultural and linguistic variety, and changes in the labour market for AI to strengthen rather than weaken socio-economic inclusion in African settings.
17.	( He et al., 2019)	• To go over some of the major practical concerns regarding the integration of AI into current clinical workflows, such as data sharing and privacy, algorithm transparency, data standardization, platform compatibility, and patient safety.	• Literature review	• Describe the current regulatory climate in the United States and draw analogies to other parts of the world, particularly China and Europe.
18.	( Hercheui & Mech, 2021)	• This paper investigates how clinicians perceive the usefulness and the ease of use of AI in healthcare. The paper aims to understand whether AI solutions are perceived to have a positive impact on patient care and the clinician’s work, and which factors affect the adoption of AI in healthcare.	• The paper draws upon key concepts of TAM (Technology Acceptance Model), adopting an exploratory approach.	• Semi-structured interviews with 22 clinicians from the NHS (the National Health System, in the United Kingdom) have provided insight into how valuable they believe AI will be for improving healthcare efficiency, quality, and diagnostic precision. • Factors like the difficulty of integrating the technology within healthcare systems (low compatibility) and understanding it (high complexity), worries about ethical issues, and the requirement for intensive training in digital skills are factors that influence how they perceive the usability of AI.
19.	( Iliashenko et al., 2019)	• To have an overview of existing cases of usage of AI in healthcare is made, and opportunities of AI technologies and challenges one may face while embedding and using them are studied.	• Statistical observation by studying sources about current projects in the AI market.	• The ability of a machine to mimic intelligent human behaviour is known as AI.
20.	( Jiang et al., 2017)	• To inspect the past, present, and past practice of AI in healthcare was the objective of the study.	• Applied survey method	• The paper revealed that cancer, neurology and cardiology are the major disease areas that use AI tools. It also stated that AI is applicable in the three major areas of early detection and diagnosis, treatment, as well as outcome prediction and prognosis evaluation of stroke.
21.	( Keskinbora, 2019)	• To discuss AI in terms of the medical ethics issues involved, both existing and potential.	• Literature review	• AI can have a wide range of beneficial applications in various fields, particularly by reducing the participation of people in extremely risky tasks. • The AI algorithms could inadvertently contain flaws that have unintended implications and biased results based on race and class.
22.	( Khullar et al., 2021)	• The objective of this study was comparing the Public and physician views of liability for AI in the healthcare ecosystem.	• The study applied survey method	• This study found that the public is significantly more likely to believe that physicians should be held responsible when an error occurs during care delivered with medical AI. It also specified that views of medical liability did not differ by clinical specialty.
23.	( Liu et al., 2020)	• To comprehend new uses for AI in medicine, including novel diagnostic techniques, metadata analysis techniques, adaptable AI-aided treatment applications in preclinical and clinical settings, as well as future perspectives of AI-aided disease prediction.	• Literature review	• Clinical medicine has made extensive use of AI, and it continues to see advancements in areas like AI-assisted lesion detection, AI-assisted image analysis, AI-assisted healthcare management, and more.
24.	( Malik et al., 2021)	• To provide a summary of potential AI model system uses in healthcare settings during the continuing Covid-19 outbreak.	• Literature review	• AI-based model systems could enhance pattern detection of disease propagation in populations and forecasts of outbreaks in various geographic regions. • By assisting public health officials in making better decisions regarding their reactions to Covid-19 cases, AI-based forecasting and predictions are anticipated to supplement conventional ways. The main concerns have been addressed with the use of AI-based methods, but the global healthcare sector has not yet seen a material change.
25.	( Matheny et al., 2020)	• To examine the benefit of AI in healthcare.	• Literature review	• The promise of AI in healthcare presents significant prospects to enhance patient and clinical team results, cut costs, and have an impact on community health. • High degrees of accuracy in imaging and signal detection tasks have been demonstrated by recent AI advances.
26.	( Mikalef et al., 2019)	• The main objective of the study was developing an AI capability framework for business value.	• The study took the concept of resource-based view of the organization as a foundation of and made a survey on existing AI literature.	• The study proposed a theoretical framework on AI capability to bring business value. It developed AI capability concepts and offered the key elements it incorporates.
27.	( Mikalef & Gupta, 2021)	• Identifying the AI-specific resources that jointly create an AI capability and providing a definition, developing an instrument to capture the AI capability of the firms, and examining the relationship between an AI capability and organizational creativity and performance were the objectives of the study.	• This study took the resource-based theory of the firm and recent work on AI at the organizational context as a lens.	• The paper publicized that AI capability marks increased organizational creativity and performance.
28.	( Naik et al., 2022)	• To address the legal and ethical issues that may arise due to the use of AI in healthcare settings.	• Literature review	• Privacy and surveillance, bias or discrimination, as well as the potential philosophical conundrum of the function of human judgment, are among the legal and ethical challenges that AI poses to society. • The introduction of modern digital technologies has given rise to worries that they may become a new source of inaccuracy and data breaches.
29.	( Nomura et al., 2021)	• To introduce AI/ML-based medical devices and prediction models regarding diabetes.	• Literature review	• Despite the current state of affairs, it is anticipated that enormous amounts of organized data and an abundance of computational resources will soon maximize the predictive performance of AI, leading to a significant increase in the accuracy of illness prediction models for diabetes.
30.	( Owoyemi et al., 2020)	• To understand the benefits of AI in healthcare.	• Literature review	• With a population of over a billion, Africa is better positioned to use AI to address its health concerns, particularly those related to maternity and child health, infectious diseases, and non-communicable diseases. • The potential that AI holds to revolutionize and advance healthcare in underdeveloped regions like Africa is quite exciting. AI implementation should focus on building intelligence into existing systems and institutions rather than attempting to start from scratch or hoping to replace existing systems. The existing use cases demonstrate that it is a viable tool for addressing health challenges, reducing costs, and improving health access and quality. • African nations must also pass laws and regulations to direct the use of this technology in healthcare and safeguard its users.
31.	( Paul et al., 2018)	• The objective of the paper was exploring the state of AI in India’s healthcare industry.	• Literature review from different sources have been applied as a method.	• The study found that AI has a range of applications across the healthcare sector by performing descriptive, predictive and prescriptive functions. It also stated that AI can augment human capacity rather than replacing human labour altogether in Indian healthcare.
32.	( Racine et al., 2019)	• To highlight potentially problematic aspects of AI use in healthcare and their effect.	• Literature review	• In domains including imaging and diagnosis, risk analysis, lifestyle management and monitoring, health information management, and virtual health aid, AI-enabled systems are being thoroughly investigated for novel healthcare applications. • Clinical methods based on AI also produce a variety of circumstances in which accepted moral standards and values may be questioned. The application of AI in healthcare may provide issues with dynamic information and consent, ownership and transparency, and privacy and discrimination. • AI-related ethical issues may give businesses a chance to develop.
33.	( Reddy et al., 2020)	• To put forth a governance model that attempts to both solve the ethical and legal concerns that result from the use of AI in healthcare and to spark more conversation on AI governance.	• Literature review	• Concerns about the moral and legal implications of using AI in healthcare include the potential for biases, the lack of transparency with some AI algorithms, privacy issues with the data used to train AI models, safety and liability concerns with AI application in clinical settings, and the possibility of biases.
34.	( Rong et al., 2020)	• The authors aimed to keep track of new scientific accomplishments, to understand the availability of technologies, to appreciate the tremendous potential of AI in bio-medicine, and to provide researchers in related fields with inspiration.	• Reviewing literature has been applied as a method. specifically, two case studies were provided to illustrate the prediction of epileptic seizure occurrences and the filling of a dysfunctional urinary bladder	• The paper showed that AI plays an increasingly important role in bio-medicine and new AI capabilities provide novel solutions for bio-medicine, and the development of bio-medicine demands new levels of capability from AI.
35.	( Rosemann & Zhang, 2022)	• To examine the practical, conceptual, and policy dimensions of the use of AI for health-related purposes from comparative and international perspectives.	• Literature review	• Concerns about the oversight, dependability, and trustworthiness of AI systems, privacy and surveillance, the impact of AI and automation on the employment of healthcare staff and the nature of clinical work, the effects of AI on health inequalities, justice, and access to medical care, as well as difficulties related to regulation and governance, are the main challenges that arise with regard to the integration of AI in medical and health care settings.
36.	( Schonberger, 2019)	• The author targets to examine the legal and ethical implications of AI in the sectors of healthcare.	• A critical analysis has been carried out as a method.	• The article provided a holistic view of AI capacities on decision making and discusses the ethical and legal consequences against the existing frameworks.
37.	( Secinaro et al., 2021)	• The objective of the study was examining the role of AI in healthcare.	• Structured literature review has been applied. The authors used qualitative and quantitative variables to analyse authors, journals, keywords, and collaboration networks among researchers.	• The research showed that the literature in the AI field is emerging, even though AI plays a great role in health. • It also stated that the United States, China, and the United Kingdom contributed the highest number of AI studies.
38.	( Shuaib et al., 2020)	• To understand AI associated challenges and benefits in healthcare.	• Literature review	• The use of AI in medicine and health care is affecting areas such as medical diagnostics, drug development, treatment personalization, supportive health services, genomics, and public health management. "AI pertains to the ability of computers or computer-controlled machines to perform activities that demand the cognitive function and performance level of the human brain. • Although AI has many benefits, its rapid adoption in the healthcare industry also raises questions about legal responsibility, morality, and data protection.
39.	( Strohm et al., 2020)	• To identify barriers and facilitators to the implementation of AI applications in clinical radiology.	• Using an embedded multiple case study, an exploratory, qualitative research design was followed.	• The pressure to control costs in the Dutch healthcare system, high expectations for the potential added value of AI, the existence of hospital-wide innovation goals, and the existence of a "local champion" were the main elements that made AI implementation easier. • The most significant barriers to the adoption of AI were the applications' inconsistent technical performance, unstructured implementation procedures, the applications' uncertain added value for clinical practice, and the wide disparity in adoption and confidence between direct adopters (radiologists) and indirect adopters (referring clinicians). • In order for AI to be successfully implemented in radiology, radiologists and referring doctors must work together. • The presence of a local champion makes it easier to implement AI in radiology. • For AI to be successfully implemented in radiology, there needs to be evidence of its clinical added value.
40.	( Tran et al., 2021)	• To develop a theoretical model to explore the behavioural intentions of medical students to adopt an AI-based Diagnosis Support System.	• This online cross-sectional survey examined the intentions to utilize an AI-based Diagnosis Support System using the unified theory of user acceptance of technology (UTAUT).	• While there was no correlation between initial trust and performance expectancy, initial trust was positively correlated with effort expectancy and social influence. The behavioural intention was only favourably correlated with social influence.
41.	Vinuesa et al. (2020)	• Assessing the effect of AI on the achievement of the sustainable development goals was the objective of the study.	• AI, sustainable development goals	• Consensus-based expert elicitation process has been applied.
42.	( Wiljer et al., 2021)	• By concentrating on the mind-sets, skill sets, and toolkits of point-of-care healthcare practitioners and their leaders within the health system, it aims to speed the appropriate adoption of data-driven and AI-enhanced treatment.	• Multi-stepped approach includes creating awareness and capacity building, learning through innovation and adoption, developing appropriate and strategic partnerships, and building effective knowledge exchange initiatives. • Education interventions • Framed by the Knowledge-to-Action framework. • An environmental scan and scoping review.	• The investigation, diagnosis, and treatment of medical diseases are being revolutionized by AI algorithms. • Clinical procedures and important health care choices are being informed by vast and complex data sets. • Fundamentals of AI, applications of AI, applied machine learning in healthcare, ethics, data science, and difficulties and opportunities for employing AI were the most often covered curricular topics in the environmental scan and scoping study.
43.	( Wolff et al., 2021)	• To bridge the gap between the notable academic AI developments of recent years and the rather meagre degree of practical use in healthcare.	• A literature and real life cases analysis was conducted in Scopus and OpacPlus as well as the Google advanced search database.	• The investigation identified three kinds of success factors: creating legislation, implementing technology, and measuring the health and financial impacts.
44.	( Vinuesa et al., 2020)	• Assessing the effect of AI on the achievement of the sustainable development goals was the objective of the study.	• Consensus-based expert elicitation processes have been applied.	• The study showed that AI can enable the accomplishment of 134 targets across all the goals, but it may also inhibit 59 targets.
45.	( Yin et al., 2021)	• The objective of the study was systematically reviewing AI applications that have been implemented in real-life clinical practice.	• This research conducted a literature search to identify relevant articles by applying inclusion and exclusion criteria.	• The study identified that AI applications targeted various clinical tasks, such as screening or triage, disease diagnosis, risk analysis, and treatment. It revealed that sepsis, breast cancer, diabetic retinopathy, and polyp and adenoma are the most commonly addressed diseases and conditions. It also indicated that despite the great potential, research on the clinical implementation of AI applications is still at an early stage.
46.	( Yu et al., 2018)	• The objective of the study was inspecting the significance of AI in the healthcare sector.	• Reviewing literature has been applied as a method.	• The study articulated that medical practice is changing through AI. It revealed that AI has implications for the economic, legal and social healthcare sectors.

### Selection process

**Figure 1.**
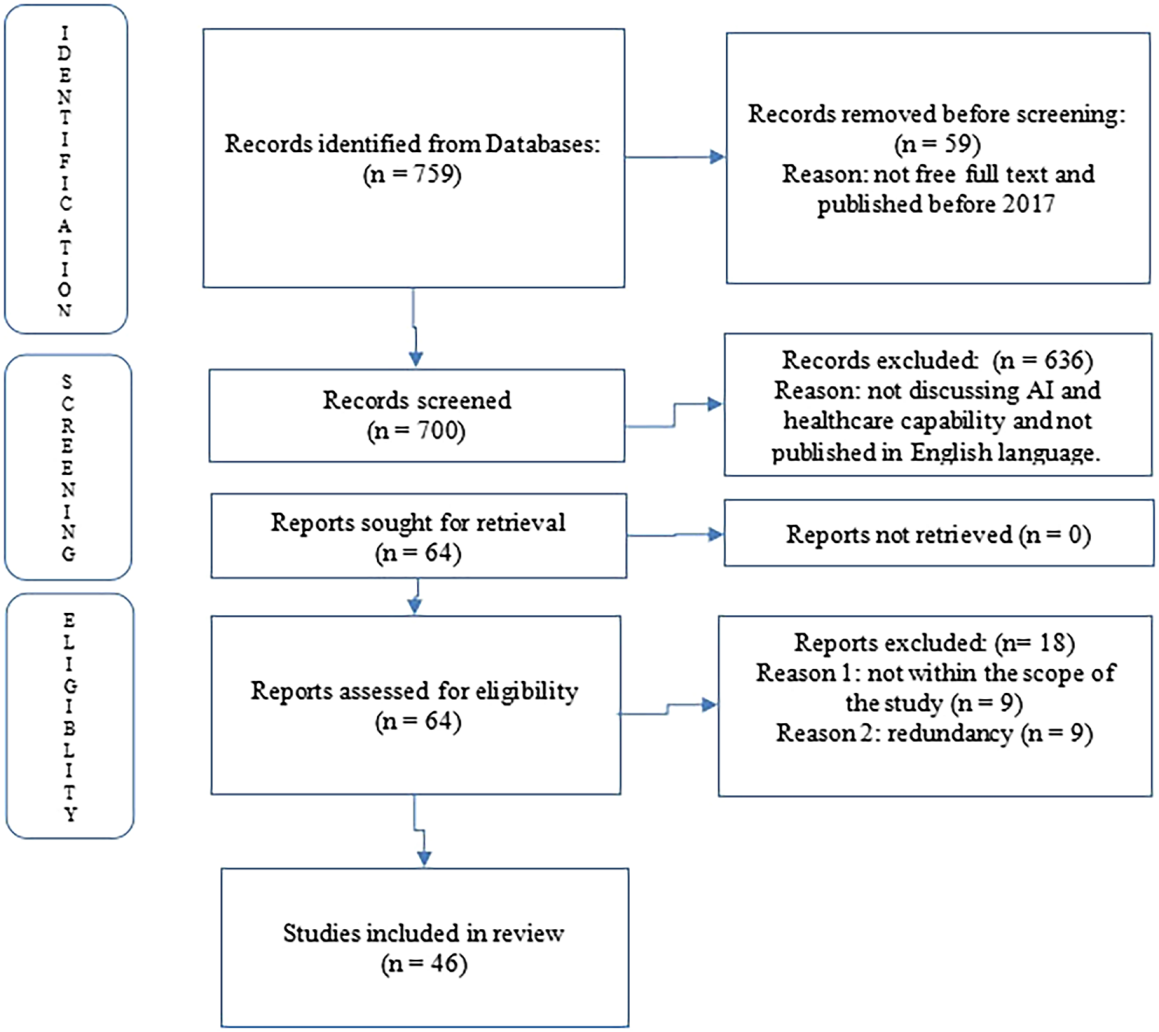
Literature review process: Author’s representation using the PRISMA approach ( Page et al., 2021).

The first step in the selection process is to find relevant articles by going through each article’s title and abstract. The complete texts of the remaining papers were examined after the abstracts to determine if they related to the current research project. The papers were also screened for duplicate research or publications, and only those that met the inclusion and exclusion requirements were downloaded and stored for later use. The bibliographies of all the remaining studies were also examined in order to locate additional published works that were not included in the online search that was chosen. Furthermore, a total of 46 papers discussing AI and healthcare capability. Following that, every chosen article was categorized under one of the main themes. As a preferred reporting item for systematic reviews, the Preferred Reporting Items for Systematic Reviews and Meta-Analysis (PRISMA) Flow Diagram in Figure 1 was adopted and used. To generate themes for this study and collect pertinent data for additional analysis, a data extraction sheet was created ( Table 1).

**Figure 2.**
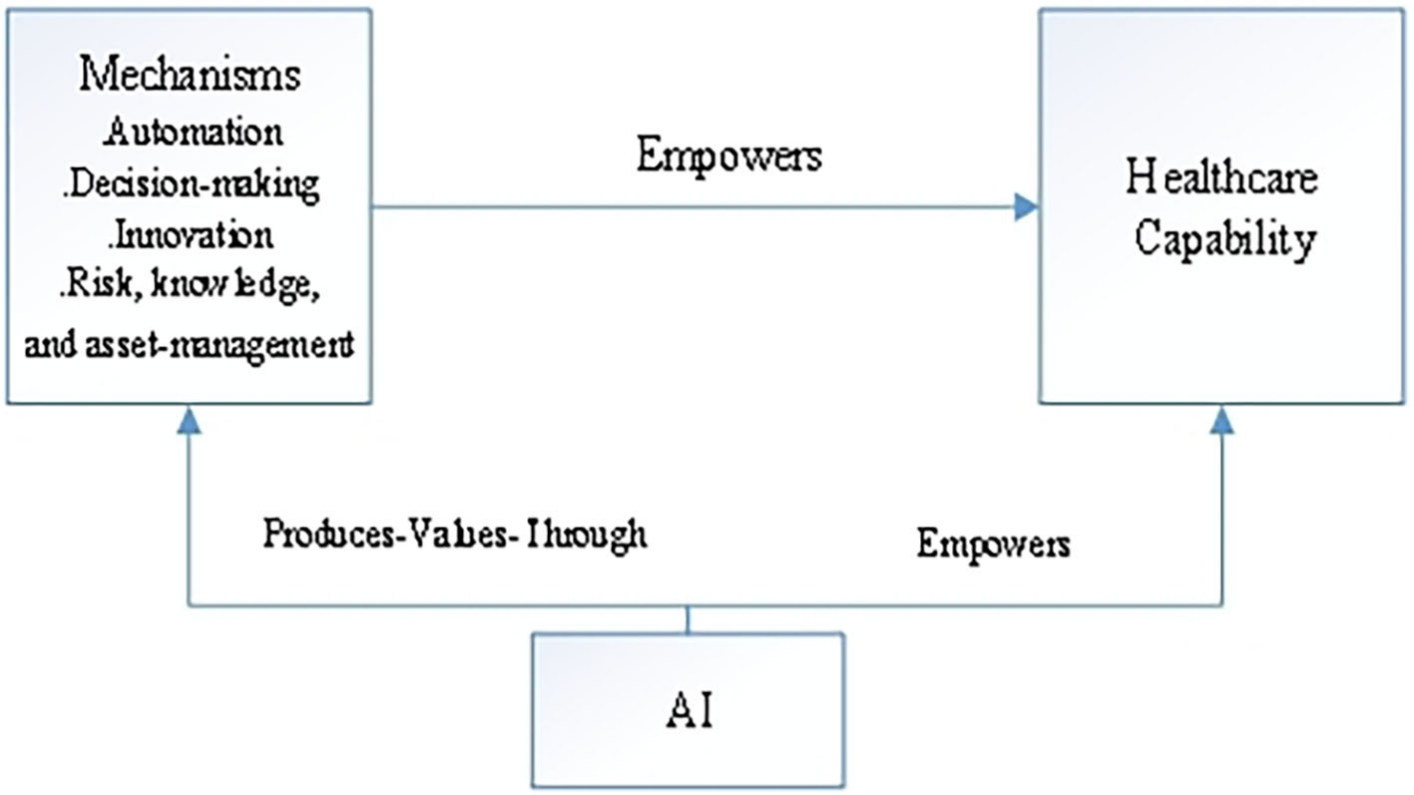
Theoretical framework (a road map for future research on AI in healthcare capability): Author’s elaboration based on existing literatures.

## Results

The PubMed search yielded 46 relevant publications after quality assessment, covering a seven-year period from 2017 to 2024. These findings underscore the critical role of AI in the healthcare industry, particularly within the context of ICT4D. AI is designed to mimic human cognitive functions ( Jiang et al., 2017) and replicate human thinking capabilities ( Mohapatra & Kumar, 2019). Its capability is defined as “the ability of a firm to select, orchestrate, and leverage its AI-specific resources” ( Mikalef & Gupta, 2021), highlighting the potential for organizations to mobilize technological innovations and optimize resources in ways that can enhance healthcare delivery in both developed and developing regions. AI is recognized as a transformative technology with the capacity to revolutionize the healthcare ecosystem ( Schonberger, 2019), creating significant enabling impacts on health outcomes ( Goralski & Tan, 2020). The integration of AI technologies is gradually transforming medical practices ( Yu et al., 2018), facilitating notable advancements in diagnostics and treatment. Additionally, AI enhances healthcare operations and delivery processes, streamlining tasks and augmenting human roles across a variety of responsibilities ( Bohr & Memarzadeh, 2020). This transformation is especially pertinent for developing economies, where AI can play a pivotal role in improving access to quality healthcare and addressing systemic challenges.

AI has the ability to drastically change healthcare and speed up medical research ( D’Antonoli, 2020). AI will continue the principal enabler and driver to the transformation of healthcare to precision medicine ( He et al., 2019). AI accomplishes healthcare enterprise management, assistance in diagnosis, and keeping a healthy lifestyle ( Iliashenko et al., 2019). AI performs treatment recommendations and diagnosis, administrative activities, adherence and patient assignation ( Davenport & Kalakota, 2019). AI solutions can enhance efficiency, healthcare quality, and diagnostic correctness ( Hercheui & Mech, 2021). Technologies in the health ecosystem such as AI can resolve gaps in quality health and reach underserved communities ( Paul et al., 2018). AI-enabled systems have the capability to advance access to quality health challenges in economically developing countries ( Kalyanakrishnan et al., 2018). AI-enabled systems play a role in diagnosis, predicting the spread of diseases and customizing treatment paths ( Secinaro et al., 2021).

AI can transform healthcare by turning big data of patients into actionable information, accelerating health responses, improving public health surveillance, and producing leaner and faster R&D ( Raghupathi & Raghupathi, 2014). Analysis, diagnosis, and treatment of medical diseases are being transformed by AI algorithms ( Wiljer et al., 2021).

Healthcare sectors show service improvement due to the recent advancement in ICT specifically e-health is emerged by the vital contribution of IT; and to improve excellent healthcare delivery systems in any country of the world, it is essential to execute solutions such as e-health ( Azeez & Van, 2019). The traditional model of medicine is completely changed due to AI technologies and this technology significantly enhanced medical services level, and assured human health in numerous features ( Liu et al., 2020). AI-enabled systems are progressively becoming an integral part of all our lives and are vital in the next-generation healthcare ecosystem ( Bohr & Memarzadeh, 2020). AI significantly impacts sectors such as public health management, genomics, medical diagnostics, treatment personalization, drug development, supportive health services ( Shuaib et al., 2020). AI has the capability to mimic human cognitive functions and can be functional to numerous categories of healthcare data such as unstructured and structured data ( Jiang et al., 2017). The expansion of novel AI systems of machine learning (ML) changed the exercise of medicine by refining diagnosis and treatment accuracy across numerous specializations ( Ahuja, 2019).

In healthcare industries, AI-enabled systems are augment physicians which are capable of caring for the upcoming medicine practice ( Ahuja, 2019). AI can help physicians by automating clinical documentation and image analysis, assisting by virtual observation, diagnosis and patient outreach ( Murali and Jayadevan, 2019). Increased health outcomes are observed using AI-based tools for many remote monitoring applications in heart failure, migraine, and diabetes management ( Jeddi & Bohr, 2020). AI-enabled systems have the capability to improve the issue of quality health in developing nations ( Kalyanakrishnan et al., 2018). AI can be applied in biomedicine because of the suitability of AI to resolve biomedical problems, and the continuous progress of AI itself ( Rong et al., 2020). The incorporation of AI-based solutions to medical services ranging from appointment scheduling via intelligent chatbots to risk profile-based insightful diagnosis, intricate surgeries guided by intelligent robots, and mentoring services that described health goals and discussed sustainable solutions towards achieving desired goals through lifestyle changes ( Guha, 2021).

In healthcare; descriptive (the most widely used which focus on event quantifying that already happened, and able to perceive trends and other insights based on the event data), predictive (it uses data from descriptive to make predictions about the future), and prescriptive (expands the purpose of predictive AI, detects trends and suggests possible treatments) are the three (3) wide groups for the uses of AI ( Paul et al., 2018). AI-based systems are also valuable in epidemiological demonstrating of Covid-19 pandemic, and to guess needs of healthcare infrastructure, human resource requirements in future when the disease spreads, that help health agencies in adopting suitable control and prevention strategies ( Malik et al., 2021). Cancer, stroke, neurology and cardiology are the major disease areas that use AI tools ( Jiang et al., 2017).

AI can enable the accomplishment of the sustainable development goals (SDGs) ( Vinuesa et al., 2020). Healthcare is one of the sectors that potentially benefited from AI ( Smith & Neupane, 2018). In terms of patient care, diagnostics, and mentoring and support services, AI has the ability to unleash a new transformation ( Guha, 2021). AI bargains significant opportunities to reduce costs, improve patient and clinical team outcomes, and stimulate people’s health ( Matheny et al., 2020), and healthcare institutions should be accountable for AI-related medical faults ( Khullar et al., 2021). AI can affect almost every aspect of the healthcare sectors from detection to prediction and prevention ( Wiljer and Hakim, 2019). AI-enabled systems practice is growing at an unprecedented speed in the healthcare industry comprising surgical operations, triage or screening, disease diagnosis, and risk analysis ( Yin et al., 2021). The health risks of patients can be identified through AI-enabled systems, as a result, AI has the potential to influence patient safety results ( Choudhury & Asan, 2020).

AI can transform the way companies do business ( Mikalef et al., 2019). AI can produce value in four different ways namely automation, decision support, marketing and innovation ( Mikalef et al., 2019). AI has a role in risk management ( Bhattacharya & Ghosh, 2007), and asset management ( Bartram et al., 2020) which can generate values for different sectors. AI can also be used to enhance the judgment and decision-making of humans in a stream termed amplified intelligence ( Zheng et al., 2017). Nowadays, AI is being deployed by many creative occupations to support innovation projects such as biomedical applications and AI is being used by designers to help in design and creativity ( Heer, 2019). Some of the studies which are focusing on AI and healthcare are presented hereunder in tabular form.

### Summary of findings

The findings revealed that AI has the potential to revolutionize the healthcare ecosystem. In recent years, there has been a significant increase in interest in integrating AI into healthcare systems, with numerous studies examining the advantages and applications of this technology. Consequently, this study identifies and analyzes literature focused on the use of AI to enhance healthcare capabilities. The researcher delimited the search by utilizing various keywords in the PubMed database to retrieve research papers published in English since 2017. The review process adhered to the PRISMA framework ( Page et al., 2021) and concentrated specifically on the intersection of AI and healthcare capability.

Based on the systematic review of 46 articles in the domain of AI and healthcare capabilities, the findings indicate that AI generates profound enabling influences on health and transforms medical practices. However, the investigation also reveals a limited understanding of how AI effectively enhances healthcare services, highlighting a nascent body of literature in this field. Following the review, the study identifies key themes emerging from the AI and healthcare literature and synthesizes these findings into a cohesive framework.

This research contributes to the body of knowledge on AI in healthcare by providing insights into the role of AI in enhancing healthcare capabilities. Additionally, it offers a framework for future empirical testing. The study underscores the need for more in-depth literature reviews and empirical research, particularly in the healthcare ecosystems of developing economies, where AI can play a crucial role in improving service delivery and addressing systemic challenges.

## Discussion

In summary, AI serves as a crucial enabler and transformative force within the healthcare sector, particularly through the lens of ICT4D (Information and Communication Technologies for Development). Despite its potential, there is a significant gap in research systematically analyzing the intersection of AI and healthcare capabilities. For instance, there remains a shortage of published studies specifically focused on AI ( Mikalef et al., 2019), while access to advanced technologies remains a challenge for many countries ( United Nations [UN], 2018). Additionally, research contributions related to business applications of AI are limited ( Bharati, 2020), highlighting the need for further exploration of AI’s role in addressing current and future health crises ( Bharati, 2020). This study addresses these gaps by examining the existing literature with a specific focus on AI and healthcare capability, aiming to elucidate how AI technologies can enhance healthcare systems, particularly in under-resourced contexts. Adopting the PRISMA framework as outlined by Page et al. (2021), the review process systematically identifies and synthesizes key themes within the literature on AI and healthcare. The findings culminate in a framework that serves as a roadmap for future research, emphasizing the critical role of AI in strengthening healthcare capabilities and promoting equitable access to health technologies globally.

## Conclusion

In conclusion, the integration of AI into healthcare is becoming increasingly essential as organizations strive to enhance their operations and decision-making capabilities amid rapid technological advancements. This study systematically addresses the existing gap in literature regarding the interplay between AI and healthcare capabilities, offering a theoretical framework that elucidates how AI can empower these capabilities. By focusing on the specific context of the Global South, the research highlights critical gaps in understanding and encourages further exploration into areas where healthcare professionals can leverage AI to improve outcomes. The study contributes to the ICT4D discourse by emphasizing the potential of AI to foster development in financially constrained environments, thereby enriching interdisciplinary dialogue around technology’s role in enhancing healthcare delivery. It provides practical insights for healthcare practitioners and policymakers in these regions, equipping them with knowledge to effectively utilize AI for better service delivery. Moreover, the research sets a foundation for empirical studies, advocating for the testing and refinement of the proposed framework within resource-limited contexts. It aims to elevate awareness among healthcare staff, managers, and technology developers about the transformative role of AI in healthcare. Given that the review is limited to literature published in English since 2017, it underscores the need for more comprehensive research that includes diverse linguistic and cultural perspectives, ultimately enriching the understanding of AI’s potential in various healthcare settings.

Dereje was a lecturer at the University of Gondar and a part-time lecturer at various institutions. He previously served as an ERP project coordinator, senior business analyst, and IT specialist in Addis Ababa. He earned a B.Sc. in Information Science from Jimma University and an M.Sc. in Information Systems from Addis Ababa University. Currently, he is a PhD candidate in Information Systems at Addis Ababa University. In 2022, he completed a Higher Diploma Program at the University of Gondar and became certified in Global Sustainable Leadership in 2024. He holds numerous certificates from prominent technology firms, including Cisco and IBM, in areas such as AI, Data Science, AWS, Linux, Agile Project Management, and Cybersecurity. He has participated in summer schools and workshops, including “Data Science 2023” at the University of Rwanda and “Climate Justice” at the University of Kenyatta. As an ambassador for Applied Machine Learning Day (AMLD) Africa-Ethiopia, he has two publications and ongoing research in Artificial Intelligence, Information Systems Security, and Digital Transformation.

## Data Availability

No data are associated with this article. Figshare: Artificial Intelligence (AI) and Healthcare Capabilities: A Systematic Literature Review. DOI: https://doi.org/10.6084/m9.figshare.27794112.v2 ( Ferede, 2024). The project contains the following reporting guidelines data:
• Extended Data Data are available under the terms of the Creative Commons Attribution 4.0 International license (CC-BY 4.0). Figshare: Artificial Intelligence (AI) and Healthcare Capabilities: A Systematic Literature Review. DOI: https://doi.org/10.6084/m9.figshare.27794112.v2 ( Ferede, 2024). The project contains the following reporting guidelines data:
•
PRISMA_2020_checklist •
Figure 1. Literature Review ProcessAuthor’s Representation Using the PRISMA Approach • Figure 2. Theoretical Framework (A Road Map for Future Research on AI in Healthcare Capability) Author’s Elaboration Based on Existing Literatures Data are available under the terms of the Creative Commons Attribution 4.0 International license (CC-BY 4.0).
