# Artificial Intelligence (AI) and Healthcare Capabilities: A Systematic Review and Research Directions

**DOI:** 10.12688/f1000research.158477.2

**Published:** 2026-04-24

**Authors:** Dereje Ferede

**Affiliations:** 1 College of Informatics, University of Gondar, Gondar, 196, Ethiopia

**Keywords:** Artificial Intelligence, Global South, Healthcare Capability, Systematic Literature Review, ICT4D, capability approach, conceptual framework, Digital health, Health information systems, ICT4D, information systems

## Abstract

Healthcare—one of the core pillars of the United Nations’ Sustainable Development Goals—is being reshaped by the rapid spread of AI. While AI adoption offers substantial opportunities for innovation, it also introduces new challenges and disrupts established healthcare practices. Hence, understanding and documenting how healthcare organizations develop AI-related capabilities and translate them into organizational value is important. This study conducts a systematic literature review to examine how healthcare organizations build and leverage AI-enabled capabilities to create value. Following the PRISMA guidelines and drawing on dynamic capability theory as the analytical framework, I conducted a structured literature search using the keywords “artificial intelligence” and “healthcare capability.” The search encompassed open-access publications from PubMed and IEEE Xplore, as well as institutionally accessed studies from the AIS eLibrary, focusing on articles published between 2015 and 2025. Applying predefined inclusion and exclusion criteria resulted in a final sample of 102 articles. I employed qualitative analysis to systematically examine the selected studies. The analysis identifies key AI tools in healthcare, their underlying micro-foundations, the AI-enabled capabilities they support, the resulting healthcare outcomes, and the challenges shaping AI-enabled healthcare. Building on these findings, I propose a process model that explains how AI tools and micro-foundations enable sensing, seizing, and transforming capabilities, which in turn drive AI-enabled healthcare outcomes. These outcomes recursively reinforce and further develop AI-enabled healthcare capabilities. This study contributes to the literature on AI and dynamic capabilities in healthcare by clarifying the mechanisms through which AI creates value. From a practical perspective, it offers actionable insights for healthcare organizations seeking to operationalize AI effectively by clarifying how AI strengthens and extends healthcare capabilities.

## 1. Introduction

Healthcare, a cornerstone of the United Nations’ Sustainable Development Goals (SDGs), is undergoing a significant transformation as artificial intelligence (AI) becomes increasingly embedded in organizational and clinical practices. Advances in machine learning, deep neural networks, natural language processing (NLP), and computer vision have enabled AI systems to analyse complex medical data, support clinical decision-making, and automate a broad range of administrative and diagnostic tasks ( Reddy et al., 2019; Yu et al., 2018). Designed to replicate and augment aspects of human cognition, AI allows healthcare organizations to process vast volumes of data and generate actionable insights at unprecedented speed and scale ( Jiang et al., 2017; Mohapatra & Kumar, 2019), thereby enhancing idea generation ( Haefner et al., 2021), improving decision quality ( Parry et al., 2016), and strengthening overall organizational performance ( Purdy & Williams, 2023). Consequently, AI applications now span clinical decision support, medical imaging, patient monitoring, personalized treatment, administrative automation, and population health management ( Davenport & Kalakota, 2019; Bekbolatova et al., 2024). These developments have given rise to AI-enabled or AI-augmented healthcare systems, in which AI plays an integrative role across clinical, operational, and governance functions ( Reddy et al., 2019).

Healthcare represents one of the most promising domains for AI deployment, particularly in light of persistent systemic challenges. These include workforce shortages and burnout, insufficient responsiveness to rising patient consumerism, slow adoption of information technologies, difficulties in assimilating increasingly complex scientific knowledge, and inadequate accommodation of patients’ evolving needs ^1^. Such pressures were further amplified by the COVID-19 pandemic, which exposed critical vulnerabilities in healthcare delivery while underscoring the need for resilient, adaptive, and data-driven systems (Vishwakarma et al., 2025). AI has demonstrated considerable potential in addressing these challenges by improving diagnostic accuracy, accelerating drug discovery, enabling telemedicine, optimizing resource allocation, and supporting real-time decision-making during public health crises ( Davenport & Kalakota, 2019; Vishwakarma et al., 2025). Beyond clinical improvements, AI contributes to operational efficiency and cost reduction by automating routine tasks, enhancing predictive analytics, minimizing unnecessary interventions, and enabling personalized care pathways ( Bekbolatova et al., 2024). AI-enabled systems have also shown promise in expanding access to quality healthcare, particularly in underserved and resource-constrained settings, thereby supporting global health equity and advancing the United Nations’ SDGs ( Paul et al., 2018; Kalyanakrishnan et al., 2018; Vinuesa et al., 2020).

Despite these opportunities, AI adoption in healthcare remains uneven and troubled with challenges. Technological issues—including data quality, interoperability, scalability, and algorithmic opacity—persist, while organizational constraints such as limited leadership commitment, skill shortages, inadequate governance structures, and resistance to change further hinder implementation ( Vishwakarma et al., 2025; Abbas et al., 2025). Ethical, legal, and social concerns—particularly related to data privacy, bias, transparency, accountability, and trust—add additional layers of complexity to AI deployment in clinical settings ( Bekbolatova et al., 2024; Choudhury & Asan, 2020). Importantly, although AI systems can match or even surpass human performance in specific tasks such as medical imaging, their limited interpretability raises concerns regarding patient trust and medico-legal accountability ( Davenport & Kalakota, 2019). Furthermore, the global healthcare sector is confronting significant challenges, including the growing complexity of diseases, a rapidly aging population, and escalating healthcare costs ( Wan, 2024).

These challenges highlight that technological superiority alone is insufficient to realize AI’s promised value in healthcare. Instead, value creation depends on healthcare organizations’ ability to purposefully select, orchestrate, and leverage AI-related resources—a capability conceptualized as AI capability ( Mikalef & Gupta, 2021). Drawing on the dynamic capabilities perspective, organizational performance is shaped not by isolated technologies but by the capacity to integrate, reconfigure, and adapt resources in response to environmental change ( Teece et al., 1997; Teece, 2007). In healthcare, this involves aligning AI technologies with clinical expertise, digital infrastructure, governance mechanisms, and patient-centered values. Recent studies reinforce the importance of adopting a capability-based perspective. For instance, De Kok et al. (2023) identify key organizational attributes that support learning and improvement in healthcare, including leadership commitment, an open culture, team development, effective change management, and strategic client focus. Similarly, Bizalwan et al. (2025) emphasize the configurational synergy among healthcare digitalization, information management, and medical AI, highlighting the critical role of stakeholder alignment in realizing sustainable AI value. Collectively, these insights suggest that focusing on organizational capabilities provides deeper explanatory power than examining individual AI applications or algorithms in isolation.

Although the literature on AI in healthcare is expanding rapidly, existing research remains fragmented and predominantly technology-centric. Prior studies mostly focus on specific algorithms, use cases, and performance metrics, offering limited insight into how healthcare organizations develop AI-enabled capabilities and translate them into sustained organizational value. While capability-oriented perspectives on AI in healthcare are increasingly recognized as important ( Zhao, 2018), further research is needed to examine how AI contributes to the SDGs while addressing potential trade-offs ( Gupta et al., 2021). In this context, research on AI for sustainability calls for the adoption of multiple value perspectives to capture both immediate outcomes and broader societal and organizational impacts ( Nishant et al., 2020). In response, this study conducts a systematic review of the literature on AI and healthcare capabilities. By shifting the analytical focus from isolated AI applications to organizational capabilities, the study advances understanding of how healthcare organizations can leverage AI as a strategic resource rather than a standalone technology. Thus, this study addresses the following research question: How do healthcare organizations build and leverage AI-enabled capabilities to create organizational value?

Guided by the PRISMA framework ( Page et al., 2021) and informed by dynamic capability theory as a sensitizing lens, I conducted a systematic search using the keywords “artificial intelligence” and “healthcare capability.” The search included PubMed and IEEE Xplore for open-access publications, as well as the AIS eLibrary via institutional access, focusing on articles published between 2015 and 2025. After applying predefined inclusion and exclusion criteria, a total of 102 articles were retained for analysis. Following the final selection, I performed a qualitative analysis using the Gioia et al. (2013) approach. This process allowed me to identify AI tools (e.g., conversational AI chatbots and agents, social robots and immersive AI systems, LLM-based generative AI systems, and deep learning–driven diagnostic AI systems) and micro-foundations (e.g., human–AI interaction and trust; governance, ethics, and organizational readiness; and data, infrastructure, and workflow integration), the resulting AI-enabled healthcare capabilities (i.e., sensing, seizing, and transforming), and their associated outcomes (i.e., clinical outcomes, patient-centered outcomes, and operational and organizational outcomes) in healthcare. Furthermore, this study identifies challenges such as trust, transparency, and professional resistance; technical and data-related limitations; and ethical, legal, and regulatory frictions. Based on these findings, I develop a process model that highlights how AI tools and micro-foundations mutually reinforce one another to enable AI-enabled healthcare capabilities—namely sensing, seizing, and transforming. Sensing and seizing capabilities interact and jointly shape transforming capabilities, which in turn drive AI-enabled healthcare outcomes. These outcomes then feedback to reinforce and further enhance the underlying capabilities, creating an iterative process. Throughout this process, AI tools, micro-foundations, capabilities, and outcomes are shaped and influenced by challenges. These insights advance the literature on AI and dynamic capabilities in healthcare by providing an understanding of how AI brings organizational value. Practically, it offers guidance for healthcare organizations seeking to adopt AI technologies effectively, highlighting the mechanisms through which AI can empower healthcare capabilities.

I organize the rest of the paper as follows. Section 2 presents the theoretical background, followed by a description of the methodology used to identify the nexus between AI and healthcare capabilities in the literature and an elaboration of the results. I then discuss the findings, outline the study’s limitations, and conclude the paper.

## 2. Theoretical Background: Dynamic Capabilities as a Sensitizing Lens

This review adopts dynamic capability theory ( Teece et al., 1997) as a sensitizing lens to examine how healthcare organizations build and leverage AI-enabled capabilities to create organizational value. This theory emphasizes that organizational performance depends not merely on technology adoption but on higher-order capabilities that allow firms to sense opportunities, seize them through strategic investments and decisions, and reconfigure resources in response to environmental change ( Teece, 2007). This perspective is particularly salient in healthcare, where AI implementation occurs within complex institutional, ethical, and operational constraints ( Davenport & Kalakota, 2019), and where the interests of multiple stakeholders must be incorporated into AI planning and deployment to ensure legitimacy, ethical alignment, and sustainable value creation ( Bizalwan et al., 2025). Framing AI as an enabling resource whose value is contingent on organizational capabilities allows this review to move beyond adoption-centric narratives and focus on the processes through which AI is translated into sustained outcomes.

Within this framework, sensing capability refers to a healthcare organization’s ability to identify, interpret, and prioritize AI opportunities relevant to healthcare delivery, such as predictive analytics for patient outcomes. In this review, sensing captures how organizations scan technological developments and evolving healthcare needs to recognize where AI can meaningfully enhance care delivery and operational performance. Seizing capability reflects the capacity to mobilize resources, redesign processes, and make strategic choices that embed AI into care pathways and organizational routines, including decisions related to investment, governance, and system integration. Reconfiguring capability denotes the ongoing adaptation of organizational assets, skills, and routines to refine AI use over time and maintain alignment with changing clinical practices, regulatory requirements, and performance objectives.

Dynamic capability research further highlights the importance of micro-foundations—the underlying skills, processes, structures, and decision rules through which dynamic capabilities are developed and enacted (Eisenhardt & Martin, 2000; Teece, 2007). These micro-foundations have been conceptualized across multiple levels, including people, structures, and processes ( Chen et al., 2023), as well as individual, organizational, and environmental dimensions ( Schilke et al., 2018). Importantly, dynamic capabilities are highly contextual ( Chatterjee et al., 2022), varying across technologies and organizational settings ( Chirumalla et al., 2023; Mikalef et al., 2021). Prior studies demonstrate the relevance of this perspective for AI-enabled value creation. For example, Mikalef et al. (2021) examine AI as an enabler of organizational capabilities, Pesqueira et al. (2025) investigate individual dynamic capabilities in healthcare operations, and Ferede and Negash (2025a, 2025b) explore AI-driven operational decision-making and transformation in different contexts. Building on this work, this review clarifies how AI-enabled capabilities are created and leveraged in healthcare settings, directly addressing the research question.

## 3. Methodology

### 3.1 Review Approach

A literature review enables scholars to address broad research questions through a holistic synthesis of existing studies, offering a comprehensive perspective on knowledge development within a focal domain ( Grant & Booth, 2009). This study examines how healthcare organizations build and leverage AI-enabled capabilities to create organizational value and is positioned at the intersection of organizing and broad theorizing reviews ( Leidner, 2018). To achieve this objective, a systematic review of studies on AI and healthcare capabilities was conducted.

Systematic literature reviews are inherently iterative and rely on the scope and quality of included studies ( Page et al., 2021). A review reporting guideline is essential for conducting a rigorous and transparent literature review, as it provides a structured framework that allows protocol modifications to be systematically documented. Following such guidelines also minimizes the risk of overlooking relevant studies. So, this study follows PRISMA ( Page et al., 2021) guidelines to structure data sources, search strategy, study selection, and analysis. The use of the PRISMA framework enhances the rigor, transparency, and reproducibility of the review, thereby strengthening the credibility and relevance of the findings. This methodological approach is particularly well suited to advancing understanding of how AI-enabled capabilities are developed and leveraged within complex healthcare settings.

Moreover, the PRISMA methodology has been widely applied in prior AI and healthcare research. For example, Abdolkhani et al. (2022) examined digital health transformation during the COVID-19 pandemic, while Choudhury and Asan (2020) investigated the role of AI in improving patient safety. Building on this foundation, the present review systematically synthesizes the literature to identify AI-enabled capabilities and their associated outcomes in the healthcare sector, thereby supporting evidence-based and equitable adoption of advanced healthcare technologies.

### 3.2 Data Source and Search Strategy

A systematic literature search was conducted using databases that are either open-access or accessible through my institution. Specifically, PubMed and IEEE Xplore are open-access, while AIS eLibrary was accessed via institutional subscription. These databases were selected for their specialization in health sciences and technology, ensuring comprehensive coverage of AI in healthcare. Additionally, the inclusion of substantial open-access content enhances accessibility for researchers in regions where subscription challenges limit access to premium resources. The literature search employed keywords aligned with the research focus, including “Artificial Intelligence” and “Healthcare Capability.” Table 1 summarizes the databases and primary keywords used in this systematic review.

**Table 1.** Search Keywords and Literature Resources.

Database Queried	Search Keywords
PubMud, IEEE Xplore, and AIS eLibrary	Artificial Intelligence and Healthcare Capability

### 3.3 Study Selection Process

This study applied a rigorous set of inclusion and exclusion criteria to ensure that the reviewed literature was relevant, current, and focused on AI applications in healthcare. Only studies published between 2015 and 2025 were considered; publications outside this timeframe were excluded. To maintain accessibility and reproducibility, only studies available as free full-text, open-access documents were included.

In terms of content, the review focused on peer-reviewed sources, specifically journal articles, conference papers, and conference series published in English. Reviews, preprints, books, and clinical trials were excluded. Studies were required to explicitly address AI applications in healthcare, while research on other technologies, such as blockchain, general digital technologies, or eHealth, was excluded.

Furthermore, the study setting was restricted to healthcare capabilities enabled by AI technologies, and participants included relevant stakeholders such as individual users, healthcare professionals, government authorities, and AI technology providers. Studies that did not involve AI-based healthcare stakeholders were excluded. Only studies whose findings contributed to a deeper understanding of AI-enabled healthcare capabilities and outcomes were considered. Table 2 summarizes the inclusion and exclusion criteria.

**Table 2.** Inclusion and Exclusion Criteria.

Criteria	Inclusion criteria	Exclusion criteria
Year of publication	2015–2025	Studies published before 2015 or after 2025
Language	English	Any language other than English
Full text availability	Free full text, open access	Not open access
Source type	Peer-reviewed publications (journal articles, conference papers, series)	Reviews, preprints, books, documents, clinical trials, randomized controlled trials
Subject area	AI applications in healthcare	Non-AI technologies (blockchain, general digital technologies, eHealth)
Study setting	AI-based healthcare capabilities	Non-AI healthcare interventions
Participants	Individual users, healthcare professionals, government authorities, AI providers	Non-AI healthcare stakeholders
Study findings	Relevant to AI-enabled healthcare capabilities and outcomes	Studies with unrelated focus

Based on these criteria, the PRISMA reporting guidelines ( Page et al., 2021) were followed, employing a four-phase review process—identification, screening, eligibility, and inclusion. In the identification phase, a total of 5,635 records were retrieved through keyword-based searches in PubMed, IEEE Xplore, and the AIS eLibrary, selected to provide comprehensive coverage of interdisciplinary research on AI and healthcare.

During the screening phase, duplicate records were removed, leaving 3,500 records. Titles and abstracts were then reviewed to exclude studies not aligned with the review focus, including those addressing non-AI technologies or systematic reviews. Only primary empirical and experimental studies explicitly examining AI in healthcare were retained, resulting in 2,956 records.

During eligibility phase, full-text assessment was conducted based on the predefined inclusion and exclusion criteria. Studies had to be peer-reviewed journal articles, conference papers, or conference series, published in English between 2015 and 2025, with full-text access. Preprints, books, clinical trials, and studies outside the specified scope were excluded. After this assessment, 95 studies met the eligibility criteria.

**Figure 1.**
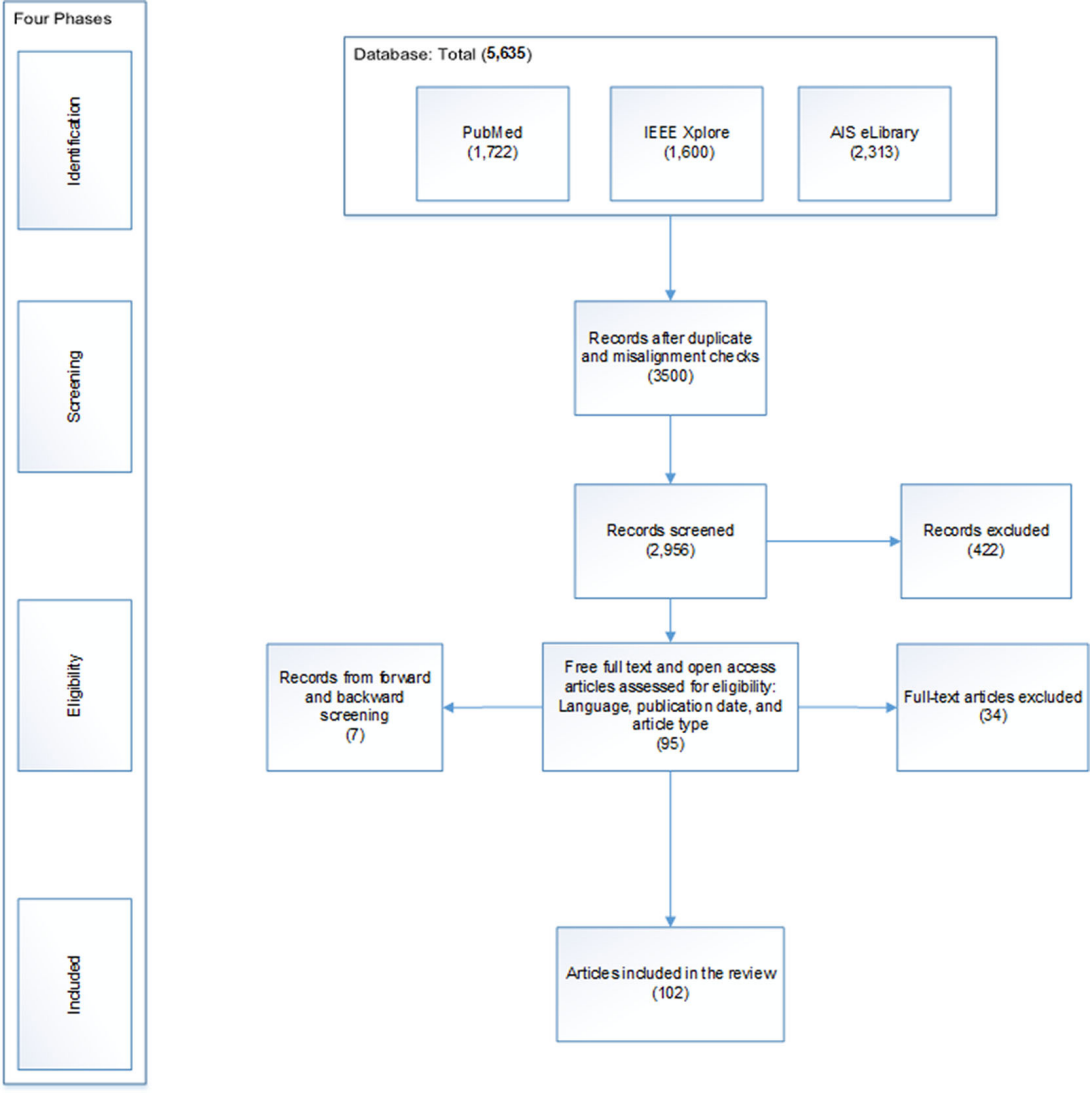
Study Selection Process in Four Phases Guided by the PRISMA Flow Diagram ( Page et al., 2021).

In the inclusion phase, additional relevant studies were identified through forward and backward citation searches of the reference lists of included articles, following Webster and Watson (2002). This process added seven studies, resulting in a final set of 102 articles. These studies collectively represent the current body of knowledge on AI and healthcare capabilities ( Figure 1).

### 3.4 Data Analysis

Data were analyzed following the Gioia et al. (2013) methodology using a manual approach in Microsoft Word. The first step involved reading all relevant articles on AI and healthcare capabilities, focusing on tools, challenges, benefits, and outcomes. Evidence from all 102 articles was incorporated into Word and reviewed in random order to identify relevant excerpts, which were iteratively assigned first-order codes through open coding. While all sections of the articles were reviewed, the coding primarily focused on the results, discussion, and conclusion sections. Open coding was guided by the objective of the study: understanding the role of AI in healthcare organizations, the challenges associated with AI adoption, and the resulting outcomes. Because articles often addressed multiple aspects of interest, multiple codes were assigned per article. First-order codes captured specific concepts in close proximity to the language used in the source documents. This process yielded the first-order codes.

Next, the analysis moved to a more interpretive phase, where second-order themes were developed. First-order codes were examined for patterns and similarities in relation to the research objectives. Similar concepts across different studies were grouped and abstracted into broader theoretical themes, such as “Conversational AI Chatbots and Agents,” “Social Robots and Immersive AI Systems,” “Human–AI Interaction and Trust Micro-foundations,” “Governance, Ethics and Organizational Readiness,” “Clinical Risk Sensing and Early Detection,” and so forth. This phase involved constant comparison of excerpts ( Glaser & Strauss, 2017), and first-order codes were refined iteratively as second-order themes emerged. This process yields the second-order themes.

Finally, relationships among the second-order themes were explored to derive seven aggregate dimensions that capture overarching patterns. Three of these—“AI-enabled Healthcare Sensing Capabilities,” “AI-enabled Healthcare Seizing Capabilities,” and “AI-enabled Healthcare Transforming Capabilities”—were aligned with dynamic capabilities theory, providing a conceptual framework for understanding how AI technologies shape healthcare organizations. For themes not adequately explained by dynamic capabilities theory, new constructs were introduced, including “AI Tools in AI-enabled Healthcare,” “AI Micro-foundations in AI-enabled Healthcare,” “AI-enabled Healthcare Outcomes,” and “Challenges in AI-enabled Healthcare,” capturing the key phenomena beyond the scope of existing theoretical frameworks.

Overall, the analysis combined inductive, abductive, and deductive reasoning. Inductive reasoning guided the development of first-order codes, abductive reasoning helped uncover relationships within the data, and deductive reasoning allowed the application of theoretical constructs from dynamic capabilities theory ( Teece et al., 1997; Teece, 2008). Table 3 visualizes the relationships among first-order codes, second-order themes, and aggregate dimensions, demonstrating how the data support the process model.

**Table 3.** Overview of the Data Structure.

First-order Codes	Second-order Themes	Aggregated Dimensions
AI-based chatbots for patient communication and appointment coordination; Conversational agents improving ED triage and telemedicine accessibility; Multilingual and culturally sensitive chatbots for diabetes care.	Conversational AI Chatbots and Agents	AI Tools in AI-enabled Healthcare
Immersive AI tools and social robots are enhancing patient engagement and reducing workflow bottlenecks.	Social Robots and Immersive AI Systems	
AI-generated patient education materials improving clarity and accessibility; LLM-assisted content structuring in online health communities; Generative AI for personalized medicine and administrative automation; LLM-powered task automation for elderly care and clinical operations; LLMs integrated with electronic health records (EHRs) for personalized patient summaries; LLMs generating CAD-RADS scores and oncology second opinions; RAG-based LLMs providing guideline-concordant recommendations.	LLM-Based Generative AI Systems	
Resource-efficient CNNs for ECG and CXR processing; MobileNet and DenseNet models for retinal diagnostics in constrained settings; deep learning systems for cancer detection and biopsy reduction.	Deep Learning–Based Diagnostic AI Systems	
Explainable AI methods using SHAP and feature attribution; Grad-CAM-based XAI for skin cancer classification; XAI-guided preprocessing for EHR data quality; AI-human hybrid systems reducing perceived judgment in mental healthcare; Anthropomorphic chatbot design enhancing empathy and engagement; Human-in-the-loop validation and adaptive oversight mechanisms.	Human–AI Interaction and Trust Micro-foundations	AI Micro-foundations in AI-enabled Healthcare
Ethical Governance and Risk Management including Ethical safeguards, bias mitigation, and privacy-aware AI deployment; LLM governance frameworks (HITL, HOTL, adaptive oversight); Organizational readiness assessments for ML adoption; Algorithm adoption shaped by IT integration, trust, and legal alignment; Capability Building and Training including AI literacy programs and simulation-based training; Clinical governance routines for expert verification; Leadership support and local innovation champions.	Governance, Ethics and Organizational Readiness	
Cloud computing, HPC, and interoperable IT systems enabling scalable AI deployment; LLM-based data extraction and OMOP standardization; Advanced ML architectures for long-term prediction; Workflow-aligned AI deployment and incremental integration strategies; Organizational learning and environmental sensing routines.	Data, Infrastructure, and Workflow Integration Micro-Foundations	
ML models detect early disease risk, deterioration, and emergency admissions; AI-based imaging for early diagnostic pattern recognition (e.g., cancer, retinal disease); Sensing patient risk factors (e.g., stroke, POST, anxiety); Population-level mental health risks sensed using multimodal data.	Clinical Risk Sensing and Early Detection	AI-enabled Healthcare Sensing Capabilities
Predictive models identify unmet care needs and preventive intervention opportunities; AI analytics identify early-stage drug candidates and disease patterns; LLMs synthesize longitudinal EHR data into actionable patient summaries; Anticipating patient volumes, workload peaks, and resource demand; Predicting patient anxiety and behavioural health needs.	AI-supported Opportunity Identification in Clinical and Operational Domains	
Organizations monitor emerging AI technologies to guide healthcare innovation; AI used to anticipate demographic, epidemiological, and resource demand trends; Assessing clinical needs and data readiness for AI adoption; Managing uncertainty across the AI lifecycle via human-in-the-loop processes; Monitoring evolving regulatory and ethical requirements; Enhancing AI explainability to support trust and adoption.	Environmental, Technological, and Governance scanning	
Risk stratification and early intervention through predictive analytics; AI-driven prioritization and real-time monitoring of care pathways; Continuous real-time patient monitoring and data-driven diagnostics; LLMs synthesizing longitudinal EHR data into actionable patient summaries.	Predictive Analytics and Proactive Care Pathway Management	
Personalizing diagnosis and treatment using AI analytics including AI-assisted individualized treatment enables precision medicine and targeted care; Hybrid deep learning models improve diagnostic accuracy in oncology; Using AI systems as clinical decision support and second opinions for example LLMs generate guideline-concordant recommendations and standardized scores; AI provides diagnostic second opinions in oncology and radiology; Generative AI automates clinical documentation and administrative workflows, reducing clinician cognitive load; LLM-based text augmentation enhances clinical NLP performance.	AI-Augmented Clinical Decision-Making	AI-enabled Healthcare Seizing Capabilities
AI tools integrated into EHRs and hospital systems enable seamless clinical use; Deployment-aware design supports operational feasibility; Clinical workflows are reconfigured to incorporate AI outputs, improving efficiency and care coordination; Predictive analytics support patient flow and resource allocation; Deploying AI-driven patient-facing systems such as Chatbots and CAIHAs support patient triage, engagement, and self-management beyond traditional encounters.	Workflow Integration and Process Reconfiguration	
Mobilizing financial, technical and human resources for AI like organizations invest in infrastructure, data capabilities, and interdisciplinary teams to enable AI adoption; Strategic AI project prioritization and stakeholder engagement accelerate adoption and value creation; Governance mechanisms ensure ethical alignment and accountability.	Resource Mobilization and Strategic AI Investment	
Early physician resistance arises from cognitive and affective concerns; Explainable AI enhances transparency and trust; AI literacy training and simulations improve clinician readiness; Hybrid human–AI decision-making reduces cognitive burden; Organizational champions and governance structures accelerate AI deployment and institutionalization.	Human–AI Collaboration and Capability Building	
Automation of administrative and operational workflows; Redistribution of cognitive workload between clinicians and AI; Iterative integration of AI insights into clinical workflows; AI-driven patient engagement and communication; Adaptive patient education and feedback mechanisms; Enhanced responsiveness to patient needs through AI.	Operational and Clinical Workflow Transformation	AI-enabled Healthcare Transforming Capabilities
Continuous retraining of AI models based on clinical feedback; Alignment of AI systems with evolving clinical guidelines; Adaptive learning loops embedded in care delivery; Reflective learning and professional development via AI feedback.	Continuous Learning and Adaptation	
Redistribution of cognitive workload between clinicians and AI; Redefinition of professional roles and identities; Reconfiguration of organizational routines to embed AI; AI-mediated interdepartmental communication and coordination; Skill and routine reconfiguration to support AI adoption; Enhancement of professional autonomy and learning.	Organizational and Professional Reconfiguration	
Explainable and interpretable AI-enabled decision support; Trust calibration shaped by cognition, context, and transparency; Human-in-the-loop oversight ensuring safety and accountability; Bias mitigation and fairness embedded in AI systems; Privacy protection and regulatory compliance mechanisms; Continuous regulatory alignment of AI applications.	Trustworthy and Responsible AI Governance	
Improved diagnostic accuracy and early intervention; Enhanced clinical decision-making and reduced diagnostic errors; Personalized treatment pathways and care planning.	Clinical Outcomes	AI-enabled Healthcare Outcomes
Enhanced patient engagement, accessibility, and time convenience; Improved patient care, personalized interventions, and PHI sharing.	Patient-centered Outcomes	
Reduced clinician workload and cognitive burden; Improved efficiency, throughput, and resource utilization; Increased clinician trust, acceptance, and confidence in AI-supported decision-making; Organizational prestige, competitive positioning, and system efficiency.	Operational and Organizational Outcomes	
Clinician skepticism toward black-box AI systems; Perceived threats to autonomy and professional identity; Cognitive workload, adoption barriers, resistance to change; Integration barriers, interoperability issues, misaligned incentives, workflow disruptions; Trust deficits, user skepticism, perceived judgment, emotional needs; Digital risks in generative AI mental health apps like technology, user perception, process; Limited interactivity, readability issues, hybrid system adoption barriers.	Trust, Transparency, and Professional Resistance	Challenges in AI-Enabled Healthcare
Data quality, bias, and missing values limit AI performance; High computational cost and scalability constraints; AI system limitations such as misclassification, false positives, accuracy, interpretability, computational efficiency, guideline alignment; Data quality issues, missing values, human labelling inconsistencies; High computational cost, limited scalability, black-box models, explainability concerns; Technical capability gaps: data availability, infrastructure, IT know-how, ethical AI deployment; Safety concerns, bias in LLMs, impact on clinical outcomes.	Technical and Data-related Limitations	
Privacy, accountability, and governance challenges; Regulatory uncertainty and ROI ambiguity hinder adoption; Legal and regulatory uncertainty, accountability issues; Ethical concerns, privacy issues, bias in AI systems; Ethical, patient-centered, and professional challenges in AI deployment; Financial constraints, cost uncertainty, ROI ambiguity.	Ethical, Legal, and Regulatory Frictions	

## 4. Results

In Version 1 of the manuscript, review articles were included in the analysis, and the findings synthesized evidence from both primary studies and review papers in accordance with the initial inclusion and exclusion criteria. In Version 2, these criteria were refined to strengthen methodological rigor, resulting in the exclusion of review articles and other non-primary sources. Accordingly, the findings are presented in two sections: Section 4.1 focuses on the Version 1 findings, which incorporate evidence from review articles, while Section 4.2 presents the Version 2 findings, reflecting the updated eligibility criteria and including only studies that met the revised standards.

### 4.1 Results: Version 1 Findings

The findings from the analyzed literature underscore the critical role of AI in the healthcare industry. AI is designed to mimic human cognitive functions ( Jiang et al., 2017) and replicate human thinking capabilities ( Mohapatra & Kumar, 2019). Its capability is defined as “the ability of a firm to select, orchestrate, and leverage its AI-specific resources” ( Mikalef & Gupta, 2021), highlighting the potential for organizations to mobilize technological innovations and optimize resources in ways that can enhance healthcare delivery in both developed and developing regions. AI is recognized as a transformative technology with the capacity to revolutionize the healthcare ecosystem (Schonberger, 2019), creating significant enabling impacts on health outcomes ( Goralski & Tan, 2020). The integration of AI technologies is gradually transforming medical practices ( Yu et al., 2018), facilitating notable advancements in diagnostics and treatment. Additionally, AI enhances healthcare operations and delivery processes, streamlining tasks and augmenting human roles across a variety of responsibilities ( Bohr & Memarzadeh, 2020). This transformation is especially pertinent for developing economies, where AI can play a pivotal role in improving access to quality healthcare and addressing systemic challenges.

AI has the ability to drastically change healthcare and speed up medical research ( D’Antonoli, 2020). AI will continue to be the principal enabler and driver of the transformation of healthcare to precision medicine ( He et al., 2019). AI accomplishes healthcare enterprise management, assistance in diagnosis, and keeping a healthy lifestyle ( Iliashenko et al., 2019). AI performs treatment recommendations and diagnoses, administrative activities, adherence, and patient assignation ( Davenport & Kalakota, 2019). AI solutions can enhance efficiency, healthcare quality, and diagnostic correctness ( Hercheui & Mech, 2021). Technologies in the health ecosystem, such as AI, can resolve gaps in quality health and reach underserved communities ( Paul et al., 2018). AI-enabled systems have the capability to advance access to quality health challenges in economically developing countries ( Kalyanakrishnan et al., 2018). AI-enabled systems play a role in diagnosis, predicting the spread of diseases, and customizing treatment paths ( Secinaro et al., 2021). AI can transform healthcare by turning big data of patients into actionable information, accelerating health responses, improving public health surveillance, and producing leaner and faster research and development ( Raghupathi & Raghupathi, 2014). Analysis, diagnosis, and treatment of medical diseases are being transformed by AI algorithms ( Wiljer et al., 2021).

Healthcare sectors show service improvement due to the recent advancement in ICT; specifically, e-health has emerged due to the vital contribution of IT, and to improve excellent healthcare delivery systems in any country of the world, it is essential to execute solutions such as e-health ( Azeez & Van der Vyver, 2019). The traditional model of medicine is completely changed due to AI technologies, and this technology significantly enhanced the medical services level and assured human health in numerous features ( Liu et al., 2020). AI-enabled systems are progressively becoming an integral part of all our lives and are vital in the next-generation healthcare ecosystem ( Bohr & Memarzadeh, 2020). AI significantly impacts sectors such as public health management, genomics, medical diagnostics, treatment personalization, drug development, and supportive health services ( Shuaib et al., 2020). AI has the capability to mimic human cognitive functions and can be functional for numerous categories of healthcare data, such as unstructured and structured data ( Jiang et al., 2017). The expansion of novel AI systems of machine learning changed the exercise of medicine by refining diagnosis and treatment accuracy across numerous specializations ( Ahuja, 2019).

In healthcare industries, AI-enabled systems augment physicians who are capable of caring for the upcoming medicine practice ( Ahuja, 2019). AI can help physicians by automating clinical documentation and image analysis, assisting by virtual observation, diagnosis, and patient outreach ( Murali & Jayadevan, 2019). Increased health outcomes are observed using AI-based tools for many remote monitoring applications in heart failure, migraine, and diabetes management ( Jeddi & Bohr, 2020). AI-enabled systems have the capability to improve the issue of quality health in developing nations ( Kalyanakrishnan et al., 2018). AI can be applied in biomedicine because of the suitability of AI to resolve biomedical problems and the continuous progress of AI itself ( Rong et al., 2020). The incorporation of AI-based solutions to medical services ranging from appointment scheduling via intelligent chatbots to risk profile-based insightful diagnosis, intricate surgeries guided by intelligent robots, and mentoring services that described health goals and discussed sustainable solutions towards achieving desired goals through lifestyle changes ( Guha, 2021).

In healthcare, descriptive (the most widely used, which focuses on event quantifying that already happened and is able to perceive trends and other insights based on the event data), predictive (it uses data from descriptive to make predictions about the future), and prescriptive (it expands the purpose of predictive AI, detects trends, and suggests possible treatments) are the three (3) wide groups for the uses of AI ( Paul et al., 2018). AI-based systems are also valuable in epidemiological demonstrations of the Covid-19 pandemic and in guessing the needs of healthcare infrastructure and human resource requirements in the future when the disease spreads, which helps health agencies in adopting suitable control and prevention strategies ( Malik et al., 2021). Cancer, stroke, neurology, and cardiology are the major disease areas that use AI tools ( Jiang et al., 2017).

AI can enable the accomplishment of the sustainable development goals ( Vinuesa et al., 2020). Healthcare is one of the sectors that potentially benefited from AI ( Smith & Neupane, 2018). In terms of patient care, diagnostics, and mentoring and support services, AI has the ability to unleash a new transformation ( Guha, 2021). AI bargains significant opportunities to reduce costs, improve patient and clinical team outcomes, and stimulate people’s health ( Matheny et al., 2020), and healthcare institutions should be accountable for AI-related medical faults ( Khullar et al., 2021). AI can affect almost every aspect of the healthcare sector, from detection to prediction and prevention ( Wiljer & Hakim, 2019). AI-enabled systems practice is growing at an unprecedented speed in the healthcare industry, comprising surgical operations, triage or screening, disease diagnosis, and risk analysis ( Yin et al., 2021). The health risks of patients can be identified through AI-enabled systems; as a result, AI has the potential to influence patient safety results ( Choudhury & Asan, 2020).

AI can transform the way companies do business ( Mikalef et al., 2019). AI can produce value in four different ways, namely automation, decision support, marketing, and innovation ( Mikalef et al., 2019). AI has a role in risk management ( Bhattacharya & Ghosh, 2007) and asset management ( Bartram et al., 2020), which can generate values for different sectors. AI can also be used to enhance the judgment and decision-making of humans in a stream termed amplified intelligence ( Zheng et al., 2017). Nowadays, AI is being deployed by many creative occupations to support innovation projects such as biomedical applications, and AI is being used by designers to help in design and creativity ( Heer, 2019).

### 4.2 Results: Version 2 Findings

Table 4 summarizes the findings and their supporting references. Analysis of the 102 reviewed articles shows that healthcare organizations are increasingly adopting AI tools while simultaneously developing the micro-foundations and AI-enabled capabilities necessary to realize AI-enabled healthcare outcomes. Table 4 further highlights both the outcomes of AI implementation and the challenges healthcare organizations face in effectively leveraging AI. A detailed synthesis of studies examining the roles of AI in healthcare is provided in the extended data while the key findings and corresponding references are consolidated in Table 4.

**Table 4.** Overview of Findings with Supporting References.

Findings		Sample References
AI Tools in AI-enabled Healthcare	Conversational AI Chatbots and Agents	( Fareedi et al., 2022, 2023; Koohikamali et al., 2025; Lai et al., 2021; Nguyen et al., 2021; Viswanath & Das, 2020)
	Social Robots and Immersive AI Systems	( Akdag et al., 2024)
	LLM-based Generative AI Systems	( Arnold et al., 2025; Balaskas et al., 2024; Chen, 2024; Khumalo et al., 2024; Liu et al., 2025; Shin & Kim, 2024; Stephenson-Moe et al., 2025; Tung et al., 2025; Wan, 2024)
	Deep Learning–Based Diagnostic AI Systems	( Anand et al., 2025; Duah et al., 2025; Mridha et al., 2023a; Shi et al., 2025; Shim et al., 2025)
AI Micro-foundations in AI-enabled Healthcare	Human–AI Interaction and Trust Micro-foundations	( Amini et al., 2024; Aslan, 2024; Mridha et al., 2023a; Prakash & Das, 2020; Tong et al., 2024; Yanes et al., 2024; Zhang et al., 2025)
	Governance, Ethics, and Organizational Readiness	( Gante & Angelopoulos, 2022; Iannantuono et al., 2024; Pumplun et al., 2021; Qureshi et al., 2024; Ringeval et al., 2025; Shi et al., 2025; Strohm et al., 2020; Zhang et al., 2025)
	Data, Infrastructure, and Workflow Integration Micro-foundations	( Adams et al., 2025; Al Reshan et al., 2023; Cheligeer et al., 2024; Esmaeilyfard & Bayati, 2025; Reis et al., 2020; Soetens et al., 2025; Strohm et al., 2020; Taherizadeh & Vaast, 2024)
AI-enabled Healthcare Sensing Capabilities	Clinical Risk Sensing and Early Detection	( Buck et al., 2021; Duah et al., 2025; Gross et al., 2021; Jacobson & Nemesure, 2021; Mridha et al., 2023b; Rahimian et al., 2018; Shi et al., 2025; Shim et al., 2025; Yadav et al., 2022; Zhou et al., 2024)
	AI-supported Opportunity Identification in Clinical and Operational Domains	( Arnold et al., 2025; Balaskas et al., 2024; Choukhi, 2024; Jacobson & Nemesure, 2021; Khumalo et al., 2024; Lou & Wu, 2019; Shim et al., 2025; Yadav et al., 2022)
	Environmental, Technological, and Governance scanning	( Aslan et al., 2022; Choukhi, 2024; Kim & Yoo, 2024; Maier et al., 2019; Raddatz et al., 2023; Sokolaj et al., 2023; Soetens et al., 2025; Taherizadeh & Vaast, 2024; Zhang et al., 2025)
	Predictive Analytics and Proactive Care Pathway Management	( Anand et al., 2025; Arnold et al., 2025; Balaskas et al., 2024; Campbell et al., 2021; Hu et al., 2025; Khojah, 2024; Shi et al., 2025; Toffaha et al., 2024; Weber et al., 2022)
AI-enabled Healthcare Seizing Capabilities	AI-Augmented Clinical Decision-Making	( Adams et al., 2025; Arnold et al., 2025; Buck et al., 2021; Han et al., 2025; Jaziri & Sassi, 2025; Khumalo et al., 2024; Latif & Kim, 2024; Simão et al., 2025; Tung et al., 2025; Wan, 2024; Yanes et al., 2024)
	Workflow Integration and Process Reconfiguration	( Akdag et al., 2024; Al-Lataifeh et al., 2024; Campbell et al., 2021; Choukhi, 2024; Gante & Angelopoulos, 2022; Koohikamali et al., 2025; Reis et al., 2020; Shim et al., 2025; Soetens et al., 2025; Strohm et al., 2020)
	Resource Mobilization and Strategic AI Investment	( Badr & Deghaim, 2024; Buck et al., 2021; Fareedi et al., 2022; Khumalo et al., 2024; Soetens et al., 2025; Zhang et al., 2025)
	Human–AI Collaboration and Capability Building	( Aslan et al., 2022; Han et al., 2025; Maier et al., 2019; Mridha et al., 2023a; Reis et al., 2020; Ringeval et al., 2025; Rosenbacke, 2024; Soetens et al., 2025; Strohm et al., 2020; Tung et al., 2025)
AI-enabled Healthcare Transforming Capabilities	Operational and Clinical Workflow Transformation	( Ashokan et al., 2025; Bass et al., 2025; Choukhi, 2024; Herschbach et al., 2025; Koohikamali et al., 2025; Lin et al., 2025; Ringeval et al., 2025; Soboleva & Mills, 2023; Stephenson-Moe et al., 2025; Strohm et al., 2020; Viswanath & Das, 2020; Weber et al., 2022)
	Continuous Learning and Adaptation	( Abdel-Karim et al., 2023; Han et al., 2025; Herschbach et al., 2025; Lin et al., 2025; Shi et al., 2025; Shim et al., 2025)
	Organizational and Professional Reconfiguration	( Badr & Deghaim, 2024; Choukhi, 2024; Koohikamali et al., 2025; Mridha et al., 2023a; Parks et al., 2023; Raj & Chandwani, 2025; Reis et al., 2020)
	Trustworthy and Responsible AI Governance	( Balaskas et al., 2024; Darvish et al., 2024; Gante & Angelopoulos, 2022; Hemmer et al., 2022; Iannantuono et al., 2024; Pumplun et al., 2021; Reis et al., 2020; Shi et al., 2025; Zhang et al., 2025)
AI-enabled Healthcare Outcomes	Clinical Outcomes	( Buck et al., 2021; Duah et al., 2025; Jacobson & Nemesure, 2021; Khojah, 2024; Mridha et al., 2023a; Shim et al., 2025; Wan, 2024; Yanes et al., 2024)
	Patient-centered Outcomes	( Akdag et al., 2024; Balaskas et al., 2024; Chaudhuri & Gupta, 2024; Raddatz et al., 2023; Viswanath & Das, 2020)
	Operational and Organizational Outcomes	( Chen, 2024; Choukhi, 2024; Han et al., 2025; Khumalo et al., 2024; Lou & Wu, 2019; Mridha et al., 2023a; Wan, 2024; Weber et al., 2022)
Challenges in AI-Enabled Healthcare	Trust, Transparency, and Professional Resistance	( Akdag et al., 2024; Gante & Angelopoulos, 2022; Khumalo et al., 2024; Koohikamali et al., 2025; Lai et al., 2021; Maier et al., 2019; Mesbah & Pumplun, 2020; Mozaffar & Cresswell, 2024; Mridha et al., 2023a; Parks et al., 2023; Prakash & Das, 2020; Raj & Chandwani, 2025; Raimi et al., 2025; Reis et al., 2020; Soboleva & Mills, 2023; Soetens et al., 2025; Stephenson-Moe et al., 2025; Strohm et al., 2020; Thimmanayakanapalya et al., 2024; Weber et al., 2024)
	Technical and Data-related Limitations	( Amini et al., 2024; Ashokan et al., 2025; Choukhi, 2024; Esmaeilyfard & Bayati, 2025; Esmaeilzadeh, 2025; Han et al., 2025; Iannantuono et al., 2024; Koohikamali et al., 2025; Lin et al., 2025; Mridha et al., 2023a; Sokolaj et al., 2023; Taherizadeh & Vaast, 2024)
	Ethical, Legal, and Regulatory Frictions	( Choukhi, 2024; Fareedi et al., 2022; Khumalo et al., 2024; Koohikamali et al., 2025; Pesonen et al., 2023; Pumplun et al., 2021; Shin & Kim, 2024; Soetens et al., 2025; Wan, 2024; Zhang et al., 2025)

The following sub-sections examine AI tools in healthcare, the micro-foundations of AI adoption, AI-enabled capabilities—sensing, seizing, and transforming—as well as the resulting outcomes and key challenges of AI-enabled healthcare.

***4.2.1 AI Tools in AI-enabled Healthcare***

The analysis reveals that healthcare organizations adopt a diverse group of AI tools to enhance clinical, operational, and patient-centered processes. These include conversational AI chatbots and agents, social robots and immersive AI systems, LLM-based generative AI systems, and deep learning–driven diagnostic AI systems. Continued interaction with these tools reshapes organizational micro-foundations, enabling clinicians and managers to develop new forms of AI-related expertise.

Conversational AI chatbots and agents are widely employed to facilitate patient communication, appointment scheduling, triage, and access to telemedicine. Many systems are multilingual and culturally sensitive, supporting chronic disease management, such as diabetes care. By automating routine patient interactions, these tools enhance engagement and reduce administrative workload in both patient-facing and back-office processes (e.g., Fareedi et al., 2022, 2023; Koohikamali et al., 2025; Lai et al., 2021; Nguyen et al., 2021; Viswanath & Das, 2020).

Social robots and immersive AI systems constitute a second category of AI tools. These technologies enhance patient engagement and support clinical workflows by addressing service bottlenecks and enabling interactive care experiences (e.g., Akdag et al., 2024). Although still emerging, they are increasingly investigated for patient interaction and care delivery applications.

LLM-based generative AI systems represent a third category, increasingly used to generate patient education materials, structure clinical documentation, and support personalized medicine and clinical decision-making. When integrated with EHRs, these systems produce actionable patient summaries, streamline administrative workflows, and facilitate evidence-based clinical decisions. Applications also include clinical decision support, knowledge synthesis, and predictive analytics (e.g., Arnold et al., 2025; Balaskas et al., 2024; Chen, 2024; Khumalo et al., 2024; Liu et al., 2025; Shin & Kim, 2024; Stephenson-Moe et al., 2025; Tung et al., 2025; Wan, 2024).

Deep learning–based diagnostic systems constitute a fourth category, frequently employing resource-efficient convolutional neural network architectures. These systems are applied to ECG analysis, chest X-ray interpretation, retinal diagnostics, and oncology detection. They enhance diagnostic accuracy, support early disease detection, and facilitate deployment in resource-constrained settings, particularly for imaging interpretation and risk stratification (e.g., Anand et al., 2025; Duah et al., 2025; Mridha et al., 2023a; Shi et al., 2025; Shim et al., 2025).

***4.2.2 AI Micro-foundations in AI-enabled Healthcare***

The adoption and effective use of AI tools in healthcare depend on organizational micro-foundations. The findings highlight three key categories: (1) human–AI interaction and trust, (2) governance, ethics, and organizational readiness, and (3) data, infrastructure, and workflow integration. These micro-foundations shape how AI tools are adopted, interpreted, and embedded into clinical and operational processes, influencing the development of AI-enabled healthcare capabilities and the sustained value these technologies deliver.

Human–AI interaction and trust represent a central micro-foundation, emphasizing explainability, transparency, and collaborative decision-making as mechanisms for trust formation. Evidence highlights the role of explainable AI (XAI) techniques—such as SHAP and Grad-CAM—in enhancing interpretability within clinical contexts, including skin cancer classification and EHR data pre-processing. Anthropomorphic chatbot designs and AI–human hybrid systems reduce perceived judgment in sensitive domains, such as mental healthcare, while human-in-the-loop validation mechanisms provide continuous oversight and adaptive calibration. Collectively, these elements underpin trust, facilitate adoption, and support effective human–AI collaboration (e.g., Amini et al., 2024; Aslan, 2024; Mridha et al., 2023a; Prakash & Das, 2020; Tong et al., 2024; Yanes et al., 2024; Zhang et al., 2025).

Governance, ethics, and organizational readiness enable healthcare organizations to adopt AI responsibly and in compliance with regulatory requirements. Key mechanisms include ethical safeguards, bias mitigation practices, privacy-aware AI deployment, and governance frameworks for LLMs, incorporating human-in/on-the-loop and adaptive oversight models. Beyond technical controls, organizational readiness—manifested through AI literacy programs, leadership engagement, systematic readiness assessments, and embedded clinical governance routines—constitutes a critical enabler for responsible AI adoption at the organizational level (e.g., Gante & Angelopoulos, 2022; Iannantuono et al., 2024; Pumplun et al., 2021; Qureshi et al., 2024; Ringeval et al., 2025; Shi et al., 2025; Strohm et al., 2020; Zhang et al., 2025).

Data, infrastructure, and workflow integration constitute the third micro-foundation, underpinning scalable and sustainable AI operations. These capabilities include cloud and high-performance computing infrastructure, interoperable IT systems, and standardized data models such as OMOP. Workflow-aligned AI deployment strategies, incremental integration approaches, and organizational learning routines further enable AI to be embedded into everyday clinical processes (e.g., Adams et al., 2025; Al Reshan et al., 2023; Cheligeer et al., 2024; Esmaeilyfard & Bayati, 2025; Reis et al., 2020; Soetens et al., 2025; Strohm et al., 2020; Taherizadeh & Vaast, 2024).

***4.2.3 AI-enabled Healthcare Sensing Capabilities***

The identified AI tools and micro-foundations reinforce one another, collectively contributing to the development of AI-enabled healthcare capabilities–sensing, seizing, and transforming capabilities. The reviewed literature indicates that healthcare organizations leverage AI-enabled sensing capabilities to identify risks, detect opportunities, and monitor emerging trends. These capabilities can be categorized into four key areas: 1) clinical risk sensing and early detection, 2) AI-supported opportunity identification in clinical and operational domains, 3) environmental, technological, and governance scanning, and 4) predictive analytics and proactive care pathway management.

Predictive analytics and proactive care pathway management represent a central sensing capability, enabling risk stratification, early intervention, and real-time monitoring of patient trajectories. Through advanced predictive modelling, AI systems prioritize care pathways, support continuous patient monitoring, and integrate longitudinal EHR data into actionable insights, ultimately enhancing care coordination and operational efficiency (e.g., Anand et al., 2025; Arnold et al., 2025; Balaskas et al., 2024; Campbell et al., 2021; Hu et al., 2025; Khojah, 2024; Shi et al., 2025; Toffaha et al., 2024; Weber et al., 2022).

Environmental, technological, and governance scanning enables organizations to monitor emerging AI technologies, demographic and epidemiological trends, regulatory changes, and evolving ethical requirements. Human-in-the-loop processes and explainability mechanisms further help manage uncertainty throughout the AI lifecycle, supporting informed strategic decision-making (e.g., Aslan et al., 2022; Choukhi, 2024; Kim & Yoo, 2024; Maier et al., 2019; Raddatz et al., 2023; Sokolaj et al., 2023; Soetens et al., 2025; Taherizadeh & Vaast, 2024; Zhang et al., 2025).

AI-supported opportunity identification in clinical and operational domains constitutes another key sensing capability. It allows healthcare organizations to proactively optimize services by identifying unmet care needs, forecasting resource demand, integrating patient data, and detecting emerging disease patterns or behavioral health risks (e.g., Arnold et al., 2025; Balaskas et al., 2024; Choukhi, 2024; Jacobson & Nemesure, 2021; Khumalo et al., 2024; Lou & Wu, 2019; Shim et al., 2025; Yadav et al., 2022).

Finally, clinical risk sensing and early detection rely on machine learning and imaging-based AI to identify emerging disease risks, track patient deterioration, and assess population-level mental health, thereby enabling timely interventions and improved outcomes (e.g., Buck et al., 2021; Duah et al., 2025; Gross et al., 2021; Jacobson & Nemesure, 2021; Mridha et al., 2023b; Rahimian et al., 2018; Shi et al., 2025; Shim et al., 2025; Yadav et al., 2022; Zhou et al., 2024).

***4.2.4 AI-enabled Healthcare Seizing Capabilities***

In addition to enhancing sensing capabilities, the reviewed studies highlight that AI strengthens seizing capabilities, defined as a healthcare organization’s ability to respond to detected opportunities or threats by mobilizing resources, reconfiguring processes, and making informed decisions. AI-enabled seizing capabilities manifest through four primary mechanisms: (1) AI-augmented clinical decision-making, (2) workflow integration and process reconfiguration, (3) resource mobilization and strategic AI investment, and (4) human–AI collaboration and capability development. Importantly, repeated seizing actions generate feedback that refines AI models, governance mechanisms, and decision rules, thereby reinforcing the organization’s sensing capabilities.

AI-augmented clinical decision-making represents a central seizing mechanism. Evidence shows that AI analytics support personalized diagnosis and treatment planning, enabling precision medicine and targeted care. Hybrid deep learning models enhance diagnostic accuracy in domains such as oncology, while LLMs are increasingly employed as clinical decision-support tools and second-opinion systems, generating guideline-concordant recommendations, standardized risk scores, and structured clinical insights. Generative AI further automates clinical documentation and administrative workflows, reducing clinician cognitive load and enabling greater focus on patient care. These capabilities collectively enhance evidence-based interventions and reduce diagnostic errors ( Adams et al., 2025; Arnold et al., 2025; Buck et al., 2021; Han et al., 2025; Jaziri & Sassi, 2025; Khumalo et al., 2024; Latif & Kim, 2024; Simão et al., 2025; Tung et al., 2025; Wan, 2024; Yanes et al., 2024).

Workflow integration and process reconfiguration constitute another key AI-enabled capability, reflecting organizations’ efforts to operationalize AI insights. AI tools are embedded in EHRs and hospital information systems to enable seamless clinical use, while deployment-aware system design ensures operational feasibility. Clinical workflows are restructured to incorporate AI outputs, improving efficiency, care coordination, and patient flow. Predictive analytics further support optimized resource allocation, and patient-facing AI applications—such as chatbots and conversational AI in healthcare (CAIHAs)—extend triage, engagement, and self-management beyond traditional clinical encounters (e.g., Akdag et al., 2024; Al-Lataifeh et al., 2024; Campbell et al., 2021; Choukhi, 2024; Gante & Angelopoulos, 2022; Koohikamali et al., 2025; Reis et al., 2020; Shim et al., 2025; Soetens et al., 2025; Strohm et al., 2020).

Resource mobilization and strategic AI investment represent a third seizing capability, enabling sustained action by aligning organizational resources with AI opportunities. Healthcare organizations invest in digital infrastructure, data capabilities, and interdisciplinary teams while prioritizing AI projects with demonstrable clinical and operational value. Strategic stakeholder engagement and governance mechanisms further ensure ethical alignment, accountability, and scalability of AI initiatives (e.g., Badr & Deghaim, 2024; Buck et al., 2021; Fareedi et al., 2022; Khumalo et al., 2024; Soetens et al., 2025; Zhang et al., 2025).

Human–AI collaboration and capability building constitute the fourth seizing mechanism, addressing cognitive, affective, and skill-related challenges. Evidence suggests that initial physician resistance—often driven by concerns over autonomy and trust—can be mitigated through explainable AI, AI literacy programs, simulation-based training, and hybrid decision-making models. Furthermore, organizational champions and robust governance structures accelerate adoption and support the long-term institutionalization of AI in clinical practice (e.g., Aslan et al., 2022; Han et al., 2025; Maier et al., 2019; Mridha et al., 2023a; Reis et al., 2020; Ringeval et al., 2025; Rosenbacke, 2024; Soetens et al., 2025; Strohm et al., 2020; Tung et al., 2025).

***4.2.5 AI-enabled Healthcare Transforming Capabilities***

Healthcare transformation through AI requires path-dependent organizational change, sustained commitment, robust governance, and continuous learning—extending beyond the mere sensing and seizing of opportunities. Evidence indicates that AI drives transformation through four interrelated mechanisms: (1) reshaping operational and clinical workflows, (2) enabling continuous learning and adaptation, (3) reconfiguring organizational structures and professional roles, and (4) supporting trustworthy and responsible AI governance. These transformative capabilities are shaped by the iterative strengthening of sensing and seizing processes.

Operational and clinical workflow transformation entails the sustained redesign of care delivery and administrative processes. AI facilitates the automation of routine tasks, redistributes cognitive workload between clinicians and systems, and integrates insights iteratively into daily clinical workflows. In addition, patient engagement and communication are enhanced through AI-driven interaction tools, adaptive education, and feedback mechanisms, enabling more responsive and personalized care (e.g., Ashokan et al., 2025; Bass et al., 2025; Choukhi, 2024; Herschbach et al., 2025; Koohikamali et al., 2025; Lin et al., 2025; Ringeval et al., 2025; Soboleva & Mills, 2023; Stephenson-Moe et al., 2025; Strohm et al., 2020; Viswanath & Das, 2020; Weber et al., 2022).

Continuous learning and adaptation capabilities enable healthcare organizations to refine AI systems and clinical practices over time. Evidence highlights ongoing retraining of AI models based on clinical feedback, alignment with evolving guidelines, and the embedding of adaptive learning loops within care delivery. AI-generated feedback also supports reflective learning and professional development among clinicians (e.g., Abdel-Karim et al., 2023; Han et al., 2025; Herschbach et al., 2025; Lin et al., 2025; Shi et al., 2025; Shim et al., 2025).

Organizational and professional reconfiguration reflects changes in roles, routines, and professional identities. AI adoption redistributes cognitive work, reshapes autonomy, and redefines clinical and managerial responsibilities. Research further documents the reconfiguration of organizational routines, AI-mediated interdepartmental coordination, and the development of skills required to embed AI into everyday practice (e.g., Badr & Deghaim, 2024; Choukhi, 2024; Koohikamali et al., 2025; Mridha et al., 2023a; Parks et al., 2023; Raj & Chandwani, 2025; Reis et al., 2020).

Trustworthy and responsible AI governance underpins transformation by ensuring ethical, legal, and safe deployment. Evidence emphasizes explainable and interpretable AI, trust calibration mechanisms, human-in-the-loop oversight, bias mitigation, privacy protection, and continuous alignment with regulatory standards. These governance practices support the sustained legitimacy and institutionalization of AI-enabled healthcare (e.g., Balaskas et al., 2024; Darvish et al., 2024; Gante & Angelopoulos, 2022; Hemmer et al., 2022; Iannantuono et al., 2024; Pumplun et al., 2021; Reis et al., 2020; Shi et al., 2025; Zhang et al., 2025).

***4.2.6 AI-enabled Healthcare Outcomes***

AI adoption in healthcare creates diverse outcomes that arise from the cumulative and reinforcing interactions of sensing, seizing, and transforming capabilities. The reviewed literature results highlight three key dimensions of impact: clinical outcomes, patient-centered outcomes, and operational and organizational outcomes.

Clinical outcomes represent one of the key values AI provides to the healthcare sector. Analysis of the reviewed studies indicates that AI implementation is associated with improved diagnostic accuracy, earlier disease detection and intervention, enhanced clinical decision-making, reduced diagnostic errors, and more personalized care pathways (e.g., Buck et al., 2021; Duah et al., 2025; Jacobson & Nemesure, 2021; Khojah, 2024; Mridha et al., 2023a; Shim et al., 2025; Wan, 2024; Yanes et al., 2024).

Patient-centered outcomes are another significant benefit of AI in healthcare. The findings further suggest that AI supports increased patient engagement, accessibility, and convenience, while enabling more personalized care, effective communication, and secure health information sharing. In particular, AI-driven interaction platforms and decision-support systems were reported to facilitate responsive, patient-focused healthcare delivery (e.g., Akdag et al., 2024; Balaskas et al., 2024; Chaudhuri & Gupta, 2024; Raddatz et al., 2023; Viswanath & Das, 2020).

Operational and organizational outcomes also demonstrate the value of AI. Evidence also indicates that AI adoption reduces clinician workload and cognitive burden, enhances operational efficiency, throughput, and resource utilization, and strengthens clinician trust in AI-supported decisions. At the organizational level, AI implementation was associated with improved institutional legitimacy, competitive positioning, and overall system efficiency (e.g., Chen, 2024; Choukhi, 2024; Han et al., 2025; Khumalo et al., 2024; Lou & Wu, 2019; Mridha et al., 2023a; Wan, 2024; Weber et al., 2022).

***4.2.7 Challenges in AI-enabled Healthcare***

Despite revealed healthcare benefits, the reviewed studies reveal persistent challenges that span AI tools, micro-foundations, capabilities, and outcomes. These challenges include 1) trust, transparency, and professional resistance; 2) technical and data-related limitations; and 3) ethical, legal, and regulatory frictions. Importantly, these challenges do not merely act as external barriers; they co-evolve with AI adoption, shaping both the development of AI-enabled healthcare capabilities and the realization of outcomes.

Trust, transparency, and professional resistance challenges encompass clinician skepticism toward black-box AI systems, perceived threats to professional autonomy and identity, increased cognitive workload, workflow disruptions, and misaligned incentives. Trust deficits are further exacerbated by limited explainability, perceived judgment in sensitive applications, and usability and emotional challenges, particularly in generative AI tools for mental health. Collectively, these challenges hinder clinician acceptance and effective human–AI collaboration (e.g., Akdag et al., 2024; Gante & Angelopoulos, 2022; Khumalo et al., 2024; Koohikamali et al., 2025; Lai et al., 2021; Maier et al., 2019; Mesbah & Pumplun, 2020; Mozaffar & Cresswell, 2024; Mridha et al., 2023a; Parks et al., 2023; Prakash & Das, 2020; Raj & Chandwani, 2025; Raimi et al., 2025; Reis et al., 2020; Soboleva & Mills, 2023; Soetens et al., 2025; Stephenson-Moe et al., 2025; Strohm et al., 2020; Thimmanayakanapalya et al., 2024; Weber et al., 2024).

Technical and data-related limitations include data quality issues, bias, missing values, labelling inconsistencies, high computational costs, scalability constraints, and limitations in accuracy and interpretability. Additionally, infrastructure gaps, safety concerns, and difficulties in aligning AI outputs with clinical guidelines—especially for LLM-based systems—have been reported (e.g., Amini et al., 2024; Ashokan et al., 2025; Choukhi, 2024; Esmaeilyfard & Bayati, 2025; Esmaeilzadeh, 2025; Han et al., 2025; Iannantuono et al., 2024; Koohikamali et al., 2025; Lin et al., 2025; Mridha et al., 2023a; Sokolaj et al., 2023; Taherizadeh & Vaast, 2024).

The review of the literature indicates that ethical, legal, and regulatory challenges encompass privacy and accountability issues, regulatory uncertainty, unclear liability structures, financial constraints, and ambiguous return on investment. These challenges complicate responsible AI deployment and introduce ongoing risks related to compliance, legitimacy, and patient trust (e.g., Choukhi, 2024; Fareedi et al., 2022; Khumalo et al., 2024; Koohikamali et al., 2025; Pesonen et al., 2023; Pumplun et al., 2021; Shin & Kim, 2024; Soetens et al., 2025; Wan, 2024; Zhang et al., 2025).

## 5. Discussion

**Figure 2.**
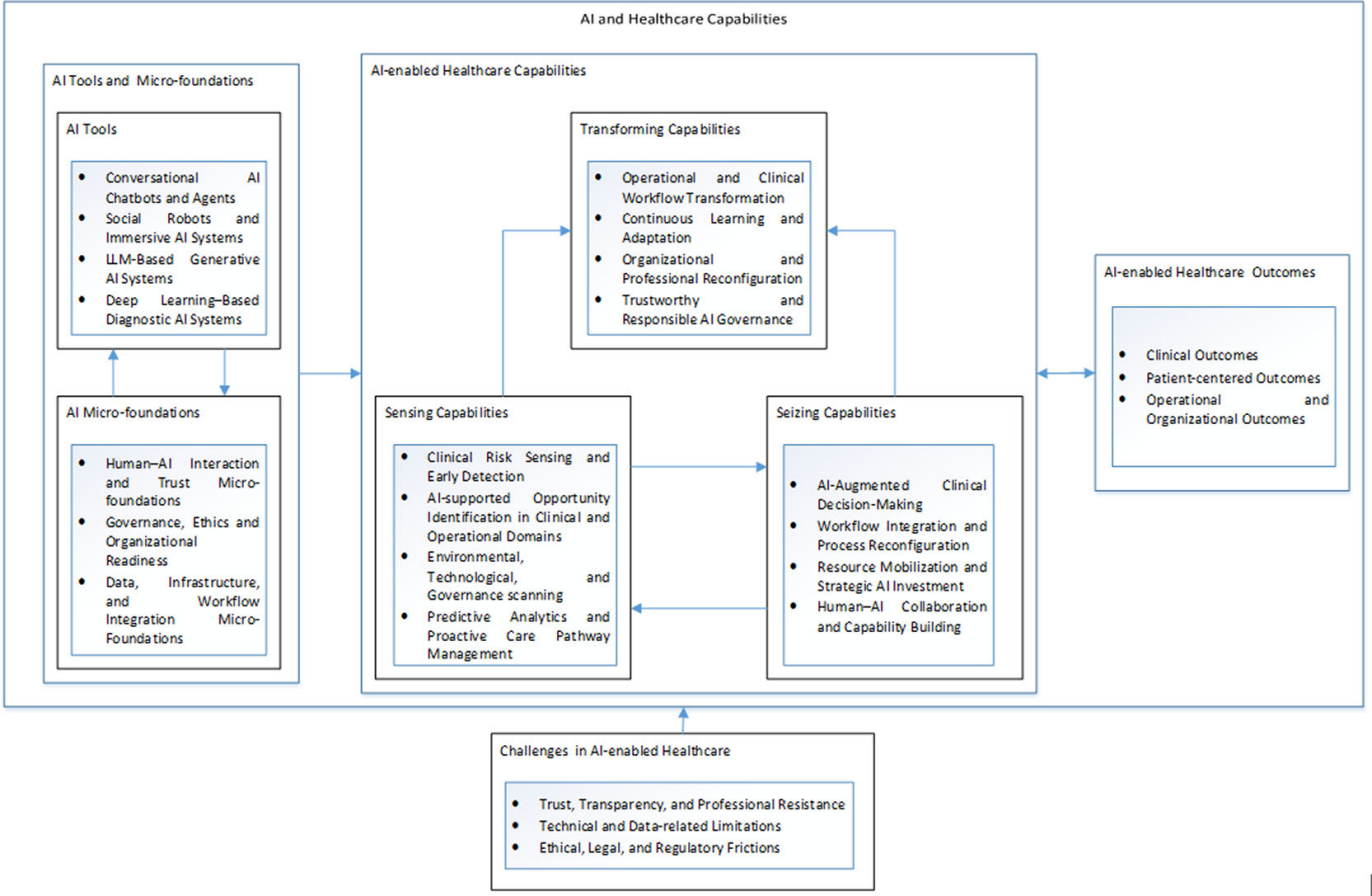
A Process Model: AI-enabled Healthcare Capabilities.

In addressing the research question—how healthcare organizations build and leverage AI-enabled capabilities to create organizational value—this study proposes a process model ( Figure 2) that positions AI-enabled value creation as an outcome of the co-evolution between AI tools and organizational micro-foundations. Rather than arising from the mere adoption of AI tools, value creation unfolds through the interplay among technology, human actors, and organizational processes. Grounded in the dynamic capability theory, the findings highlight how AI enables healthcare organizations to sense opportunities and threats, seize value-creating initiatives, and transform existing routines in response to environmental uncertainty, while simultaneously navigating the associated challenges.

The findings indicate that while the adoption of AI tools—ranging from conversational chatbots and social robots to LLM-based generative AI and deep learning diagnostic systems—is necessary for AI-enabled healthcare transformation, it is not sufficient on its own to drive capability development and value creation. Without alignment with organizational structures, professional roles, and governance systems ( Ahuja, 2019; Wiljer et al., 2021; Shuaib et al., 2020), AI adoption alone is unlikely to generate meaningful clinical, patient-centered, or operational outcomes. Consistent with a dynamic capabilities perspective ( Teece et al., 1997; Teece, 2007), the effectiveness of AI depends on complementary organizational micro-foundations that enable organizations to select, orchestrate, and leverage AI-specific resources ( Mikalef & Gupta, 2021). These micro-foundations include human–AI interaction mechanisms, trust-building practices, data governance structures, workflow integration routines, organizational readiness, and ethical oversight. The findings highlight that mechanisms such as explainable AI, human-in-the-loop decision processes, clinician training, and anthropomorphic chatbot design are critical for translating AI-generated outputs into actionable insights and supporting trusted clinical decision-making. In line with Chen et al. (2023), these micro-foundations are dynamic: they evolve through experimentation, learning, and feedback from clinical practice. AI tools and organizational micro-foundations therefore reinforce each other. AI applications can stimulate the development of new governance practices, skill enhancement, and workflow redesign, while micro-foundations enhance the deployment, acceptance, and refinement of AI tools. This reciprocal relationship underpins the development of AI-enabled healthcare capabilities–sensing, seizing, and transforming. Based on these insights, I provide the following proposition.

Proposition 1. In the healthcare context, AI-enabled capabilities—sensing, seizing, and transforming—emerge from the mutual reinforcement between AI tools and organizational micro-foundations, including human–AI interaction mechanisms, trust-building practices, data governance, workflow integration, organizational readiness, and ethical oversight, rather than from AI adoption in isolation.

The model highlights that AI-enabled healthcare capabilities are most prominently expressed through the processes of organizational sensing, seizing, and transforming. AI significantly enhances sensing capabilities by enabling large-scale data integration, early risk detection, and pattern recognition across clinical, operational, and population health domains. These improvements allow healthcare organizations to anticipate patient needs, identify emerging public health trends, and detect operational bottlenecks. At the same time, AI strengthens seizing capabilities by supporting evidence-based interventions, workflow optimization, predictive analytics, and proactive care pathway management. Importantly, the findings suggest that sensing and seizing are not sequential stages but mutually reinforcing processes. Enhanced sensing allows for faster, more targeted interventions, while AI-supported seizing generates new data, performance feedback, and organizational learning that further refine sensing. This recursive feedback loop reflects AI’s capacity to transform large volumes of heterogeneous healthcare data into actionable insights, accelerating clinical response, improving public health surveillance, and enhancing research efficiency ( Raghupathi & Raghupathi, 2014). By shortening learning cycles and improving organizational responsiveness under uncertainty, the interplay between sensing and seizing contributes to the development of transforming capabilities, as evidenced in predictive diagnostics, early detection, and patient monitoring applications ( Jeddi & Bohr, 2020; Malik et al., 2021). These lead us to the second proposition.

Proposition 2. Within AI-enabled healthcare environments, the mutual reinforcement of sensing and seizing capabilities drives the emergence of transforming capabilities.

Unlike sensing and seizing, transforming capabilities develop more slowly and reflect deeper and path-dependent organizational change. Transforming involves the reconfiguration of clinical workflows, the redefinition of professional roles, the institutionalization of AI into routine practices, and the alignment of AI use with ethical, regulatory, and cultural norms. This temporal asymmetry underscores the importance of leadership commitment, governance structures, and organizational culture—attributes closely aligned with learning and improvement capabilities in healthcare, such as leadership commitment, open culture, team development, and strategic client focus ( De Kok et al., 2023). While early gains from AI adoption—such as efficiency improvements and predictive insights—may be realized relatively quickly, sustained transformation requires continuous investment in human–AI collaboration, professional learning, and iterative organizational adaptation. Beyond operational efficiency, AI’s transformative potential extends to reshaping interprofessional coordination, embedding evidence-based practices, and redefining accountability structures within healthcare organizations ( Ahuja, 2019; Guha, 2021). Based on these insights, I propose the following proposition.

Proposition 3. AI-enabled healthcare transforming capabilities develop more slowly than sensing and seizing capabilities due to their dependence on governance, leadership commitment, and cultural alignment.

Consistent with prior studies ( Wiljer & Hakim, 2019; Matheny et al., 2020; Yin et al., 2021), the findings suggest that AI does not directly improve healthcare outcomes. Rather, AI tools and their underlying micro-foundations enhance intermediate AI-enabled healthcare capabilities—sensing, seizing, and transforming—which in turn drive improvements in clinical, patient-centered, and operational outcomes. In other words, AI-enabled outcomes such as diagnostic accuracy, personalized care, operational efficiency, patient engagement, and organizational learning ( Kalyanakrishnan et al., 2018; Jeddi & Bohr, 2020; Rong et al., 2020) emerge from the development of these capabilities. Importantly, this relationship is bidirectional. As outcomes improve, they create new demands for AI-enabled capabilities. For example, advances in personalized care increase the need for sophisticated data integration and predictive analytics, while gains in operational efficiency raise expectations for scalability and interoperability. This reciprocal dynamic underscores that AI-enabled healthcare outcomes are both the product of and a driver for continuous capability development. These insights lead to the following proposition:

Proposition 4. In healthcare settings, the development of AI-enabled capabilities is essential to achieving AI-enabled healthcare outcomes, which in turn create a positive feedback loop that both drives and demands further advancement in sensing, seizing, and transforming capabilities.

Furthermore, the findings indicate that challenges in AI-enabled healthcare—such as trust deficits, transparency concerns, professional resistance, technical and data limitations, and ethical, legal, and regulatory frictions—extend beyond merely constraining AI adoption. These challenges co-evolve with AI tools, micro-foundations, healthcare capabilities, and outcomes, actively shaping their development and use. For instance, poor data quality can undermine diagnostic AI performance, prompting investments in data governance, infrastructure, and clinician training. These responses, in turn, strengthen micro-foundations and refine clinical workflows. Managing these co-evolving challenges is therefore critical for sustaining AI adoption and achieving long-term healthcare outcomes ( Khullar et al., 2021). Thus, my fifth proposition is:

Proposition 5. AI tools, micro-foundations, healthcare capabilities, and outcomes are shaped by challenges related to trust, transparency, technical and data constraints, and ethical, legal, and regulatory frictions in the healthcare context.

### 5.1 Implications for Theory and Practice

Theoretically, this study contributes to scholarship on AI, dynamic capabilities, and healthcare transformation by responding to two calls in prior research. First, while existing studies highlight the strategic potential of AI, they offer limited insight into how organizations develop capabilities and generate value from AI in practice ( Zhao, 2018; Mikalef et al., 2019). Second, scholars have emphasized the need to examine AI’s role in sustainable value creation and broader societal outcomes ( Gupta et al., 2021; Nishant et al., 2020), including healthcare performance and patient well-being. As such, this study contributes to the literature in four interrelated ways.

First, it advances research on AI in healthcare by shifting from technology-centric explanations of value creation (Cheng et a., 2011) to a capability-focused perspective ( Teece et al., 1997; Teece, 2008). Rather than viewing AI as a direct driver of healthcare outcomes, this study highlight that value emerges through AI-enabled capabilities—sensing, seizing, and transforming ( Propositions 1–4). By framing AI adoption as an organizational learning and capability-building process, the study show how AI interacts with micro-foundations such as workflows, governance structures, and professional routines. This approach provides a more nuanced understanding of how AI creates clinical, operational, and patient-centered value.

Second, the study extends dynamic capability theory into AI-enabled healthcare by adopting a micro-foundational and process-oriented lens. The study explicate how AI tools interact with organizational micro-foundations—managerial cognition, clinical routines, data governance practices, and cross-functional collaboration—to reveal the mechanisms through which sensing, seizing, and transforming capabilities emerge and co-evolve over time. Notably, the findings highlight temporal asymmetries: transforming capabilities evolve more slowly and contentiously than sensing and seizing capabilities. This insight enriches dynamic capability theory by clarifying temporal dynamics in AI-intensive healthcare contexts.

Third, this research advances theorizing on healthcare transformation by conceptualizing AI-enabled change as a recursive, path-dependent, socio-technical process rather than a linear adoption process. Challenges such as data quality limitations, clinician resistance, and ethical and regulatory constraints are not merely barriers; they co-evolve with AI tools, micro-foundations, capabilities, and outcomes ( Proposition 5). Viewing AI adoption as recursive and path-dependent deepens understanding of healthcare transformation and highlights opportunities for iterative learning and adaptation.

Fourth, by linking AI tools, micro-foundations, AI-enabled capabilities, outcomes, and challenges within a unified framework, this study provides a process model for AI-enabled healthcare transformation. This model clarifies how sensing, seizing, and transforming capabilities interact over time and underscores the importance of addressing challenges as integral to capability development.

Practically, this study offers several important implications for key stakeholders involved in the development and deployment of AI in healthcare. First, implications for policymakers and regulators. The findings suggest that policymakers should emphasize capability-building rather than focusing narrowly on technology acquisition. Public funding and policy initiatives would be more effective if they prioritized workforce development, robust data governance, and organizational learning processes alongside investments in AI technologies. Regulatory frameworks should remain adaptive, enabling controlled experimentation while maintaining safeguards for safety, equity, and accountability. Furthermore, equitable AI deployment requires deliberate attention to disparities in access, data representation, and health outcomes, ensuring that AI-enabled innovation aligns with broader public health objectives.

Second, for AI developers and vendors, the results underscore the importance of designing systems that can be flexibly integrated into diverse clinical contexts. Rather than imposing rigid workflows, AI solutions should support the evolution of existing organizational routines. Long-term partnerships with healthcare organizations—extending beyond initial deployment to include training, governance support, and ongoing capability development—are critical for sustained value creation. Embedding transparency through explainability, auditability, and ethical safeguards can further enhance trust, facilitate organizational adoption, and support compliance with emerging regulatory requirements.

Third, implications for clinicians and healthcare professionals. The study highlights the role of AI as a capability amplifier rather than a substitute for clinical expertise. Clinicians can leverage AI to enhance diagnostic accuracy, decision-making, and continuous learning while maintaining professional autonomy. Early involvement of clinicians in co-design and implementation processes is particularly important, as it fosters trust, improves system relevance, and accelerates both individual and organizational capability development. In addition, continuous investment in data and AI literacy is essential for integrating AI insights meaningfully into clinical practice.

Fourth, implications for healthcare organizations. At the organizational level, the findings reinforce the need to view AI as a strategic toolkit whose value depends on complementary organizational capabilities rather than on technology adoption alone ( Jarrahi, 2018). Leaders and managers should therefore shift their investment logic from tools to capabilities, evaluating AI initiatives based on their contribution to organizational effectiveness and learning rather than short-term financial returns. AI projects should be embedded within broader capability development strategies and sequenced deliberately, progressing from pilot applications that enhance situational awareness to initiatives that enable deeper healthcare transformation. Governance structures and organizational culture should be treated as strategic assets, with strong data governance, ethical oversight, and leadership commitment institutionalized early. Lastly, investments in micro-foundations—such as workforce skills, interdisciplinary routines, and clinical–technical collaboration—along with explainable AI mechanisms can support effective human–AI collaboration and build sustained organizational trust.

## 6. Limitations, Research Directions, and Conclusion

### 6.1 Limitations

Despite its contributions to understanding AI-enabled healthcare capabilities, this study has limitations. First, the review was confined to peer-reviewed articles published in English. Consequently, non-English publications were excluded. While this restriction enhanced methodological rigor and reliability, it may have limited the inclusion of perspectives from regions where AI-enabled healthcare innovation is rapidly advancing but less frequently documented in English-language journals.

Second, the choice of academic databases may have influenced the scope of the review. Although major databases were systematically searched to ensure coverage of high-quality research, relevant studies indexed in alternative sources may not have been captured. Accordingly, the findings should be interpreted in light of the selected databases and search strategy, and future reviews could broaden database coverage to validate and extend these insights.

Third, the rapid evolution of AI technologies represents an inherent limitation. Advances in machine learning techniques, data infrastructures, and regulatory frameworks may outpace the evidence captured in this review. As a result, some findings may reflect transitional stages of AI-enabled capability development rather than stable or long-term patterns.

Fourth, this study adopts dynamic capability theory as its guiding analytical framework. While this perspective is particularly well suited to examining how healthcare organizations develop and leverage AI-enabled capabilities, the resulting insights are shaped by this theoretical lens and might differ if alternative frameworks were applied.

Fifth, consistent with grounded theory–informed approaches (e.g., Gioia et al., 2013), data interpretation involves a degree of subjectivity. To enhance transparency and credibility, the complete data structure is provided in Table 3, allowing readers to assess the analytical process.

Finally, as with all systematic reviews, the insights generated depend on the quality, depth, and theoretical orientation of the included studies. Although this review advances theory by synthesizing and reinterpreting prior research through a dynamic capability lens, it does not offer direct empirical validation of the proposed relationships.

### 6.2 Research Directions and Future Agenda

Building on these limitations, several avenues for future research emerge. First, while conceptual studies emphasize the potential of AI to enhance healthcare delivery, empirical evidence remains limited regarding how specific AI capabilities translate into distinct healthcare system capabilities. Future research should employ quantitative, qualitative, and mixed-methods designs to examine how capabilities such as predictive analytics, computer vision, and NLP influence diagnostic, decision-making, operational, learning, and public health capabilities across diverse healthcare contexts. Key questions include how different AI capabilities affect diagnostic accuracy and clinical decision-making, which capabilities most strongly enhance operational efficiency and care coordination, and how complementarities among AI capabilities shape overall healthcare performance.

Second, attention should be directed toward AI-enabled capability development in low- and middle-income countries, where contextual conditions and non-English scholarship remain underrepresented. Healthcare systems in these settings face distinct infrastructural, institutional, and workforce constraints that may alter capability development pathways. Integrating ICT4D, institutional, and comparative perspectives could illuminate how AI addresses workforce shortages and infrastructure gaps, the regulatory and cultural barriers shaping AI adoption, and how resource constraints influence sensing, seizing, and transforming capabilities.

Third, most of the existing research assesses AI value through outcome-based indicators such as cost reduction and clinical accuracy, offering limited insight into the organizational capabilities underpinning long-term transformation. Future studies should develop capability-oriented measurement frameworks that capture intermediate outcomes, including organizational learning, adaptability, resilience, and equity of access. Such metrics may provide a more accurate basis for predicting sustained system-level performance.

Fourth, governance, ethics, and accountability warrant examination not merely as compliance requirements but as enabling micro-foundations of AI-driven capability development and trust. Future research should explore how regulatory environments and data governance frameworks shape AI-enabled sensing and seizing capabilities, which governance mechanisms foster transparency and accountability, and how ethical AI practices such as explainability and fairness influence clinician acceptance and capability accumulation.

Fifth, the predominance of cross-sectional studies and analyses of isolated AI applications limits understanding of the dynamic and path-dependent nature of AI-enabled transformation. Longitudinal, process-oriented, and system-level research is needed to examine how capabilities co-evolve over time and how AI reshapes healthcare ecosystems. Questions include how sensing, seizing, and transforming capabilities develop with sustained AI use and why transforming capabilities often lag behind earlier stages.

Sixth, as AI increasingly complements rather than replaces healthcare professionals, future research should examine how human–AI collaboration reshapes roles, skill requirements, and organizational routines. Investigating how trust, explainability, and usability influence human–AI interaction can deepen understanding of capability development at both individual and organizational levels.

Finally, research should address the broader societal and sustainability implications of AI-enabled healthcare, particularly in relation to universal health coverage, health equity, and the SDGs. Examining how AI-enabled capabilities support preventive care, reduce health inequalities, and contribute to inclusive and sustainable development represents a critical future agenda.

## 7. Conclusion

This study advances understanding of AI-enabled healthcare transformation by adopting a capability-based perspective that moves beyond technology-centric explanations. Through a systematic review, it highlights that AI creates value not through adoption alone, but through its integration with organizational micro-foundations and AI-enabled healthcare capabilities. AI strengthens sensing, seizing, and transforming processes by enabling data-driven insights, informed decision-making, and workflow reconfiguration, with value emerging indirectly through enhanced intermediate capabilities that support improved clinical, operational, and patient-centered outcomes. Challenges related to data quality, trust, and regulations co-evolve with these tools, micro-foundations, capabilities, and outcomes, shaping their effectiveness over time. Overall, the findings position AI as a strategic enabler whose impact depends on the interplay of technology, people, processes, and organizational learning. In conclusion, continued scholarly, policy, and managerial efforts are required to fully realize the transformative potential of AI in healthcare.

Dereje Ferede is a lecturer at the University of Gondar. He has previously held roles as a senior business analyst, ERP coordinator, and information systems specialist. His research interests encompass digital policy, artificial intelligence, digital transformation, and information systems security.

## Data Availability

No data are associated with this article. Figshare: Artificial Intelligence (AI) and Healthcare Capabilities: A Systematic Review and Research Directions. DOI: 10.6084/m9.figshare.31338331 ( Ferede, 2026). The project contains the following reporting guidelines data:
• Extended Data Data are available under the terms of the Creative Commons Attribution 4.0 International license (CC-BY 4.0). Figshare: Artificial Intelligence (AI) and Healthcare Capabilities: A Systematic Review and Research Directions. DOI: 10.6084/m9.figshare.31338331 ( Ferede, 2026). The project contains the following reporting guidelines data:
• Summary of Reviewed Literatures •
PRISMA_2020_checklist Data are available under the terms of the Creative Commons Attribution 4.0 International license (CC-BY 4.0).

## Footnotes

^1^

https://www.ncbi.nlm.nih.gov/books/NBK221522/
