# Supplementary material for: Artificial Intelligence (AI) and Healthcare Capabilities: A Systematic Review and Research Directions
Source: F1000Res. 2026 Apr 24;14:20. Originally published 2025 Jan 3. [Version 2] doi: 10.12688/f1000research.158477.2 (PMC13197750; doi:10.12688/f1000research.158477.2)
Supplement: Peer review history

# Reviewer report: Reviewer response for version 2

**DOI:** 10.5256/f1000research.195995.r485391

**Published:** 2026-05-22

**Authors:** Shehu Mohammed

**Affiliations:** 1 Lovely Professional University, Pagwara, Punjab], India

This revised manuscript presents a systematic review on AI-enabled healthcare capabilities using dynamic capability theory as its conceptual lens. Compared with the earlier version, the manuscript has improved considerably. The author has addressed several major concerns raised previously, including broader literature coverage, improved methodological description, stronger theoretical framing, expanded discussion of ethical and governance issues, and clearer practical implications.

 The study addresses a relevant and timely topic, and the rationale and objectives are clearly stated. The conclusions are generally supported by the findings presented.

 However, some methodological concerns remain. Although the methodology section is much improved, the review is only partially reproducible because key details such as exact search strings, search dates, duplicate handling, and validation of the screening/coding process are not fully transparent. In addition, the absence of a formal quality assessment of included studies limits the strength of the evidence synthesis. Database coverage has improved, but the exclusion of other major databases may still affect comprehensiveness.

 Overall, this is a substantially improved manuscript that makes a useful contribution, but some methodological limitations remain.

Are the rationale for, and objectives of, the Systematic Review clearly stated?

Yes

Is the statistical analysis and its interpretation appropriate?

Not applicable

If this is a Living Systematic Review, is the ‘living’ method appropriate and is the search schedule clearly defined and justified? (‘Living Systematic Review’ or a variation of this term should be included in the title.)

Not applicable

Are sufficient details of the methods and analysis provided to allow replication by others?

Partly

Are the conclusions drawn adequately supported by the results presented in the review?

Yes

Reviewer Expertise:

AI in healthcare, machine learning, health technologies, and health informatics.

I confirm that I have read this submission and believe that I have an appropriate level of expertise to confirm that it is of an acceptable scientific standard, however I have significant reservations, as outlined above.

# Reviewer report: Reviewer response for version 2

**DOI:** 10.5256/f1000research.195995.r478879

**Published:** 2026-05-09

**Authors:** Ferry Fadzlul Rahman

**Affiliations:** 1 Universitas Muhammadiyah Kalimantan Timur, Samarinda, Indonesia

The revised manuscript demonstrates substantial improvement in terms of conceptual clarity, theoretical framing, and methodological transparency. The integration of dynamic capability theory provides a stronger analytical lens for understanding how AI-enabled capabilities are developed and transformed within healthcare organizations. The revised abstract also presents a clearer narrative flow, beginning from the healthcare context and progressing logically toward the study’s contribution. The inclusion of a process model linking AI tools, micro-foundations, dynamic capabilities, and healthcare outcomes significantly strengthens the manuscript’s theoretical contribution.

 The author has also improved the methodological description by explicitly stating the use of PRISMA guidelines, identifying the databases searched, clarifying the publication period (2015–2025), and describing the inclusion and exclusion process that resulted in 102 selected studies. This revision improves transparency and reproducibility compared to the earlier version. In addition, the focus on AI-enabled healthcare capabilities rather than solely AI applications enhances the originality and strategic relevance of the study.

 Another strength of the revision is the clearer articulation of practical implications. The manuscript now better explains how healthcare organizations can operationalize AI through sensing, seizing, and transforming capabilities. This alignment between theory and practice increases the manuscript’s value for both academic and managerial audiences.

 Despite these improvements, several areas could still be strengthened before indexing. First, although the manuscript mentions qualitative analysis, the analytical procedure remains somewhat general. The authors should further clarify how themes or categories were derived from the reviewed studies, including whether coding procedures, thematic synthesis techniques, or reviewer triangulation were applied. This would strengthen methodological rigor.

 Second, the database selection could be discussed more critically. While PubMed, IEEE Xplore, and AIS eLibrary are relevant sources, the exclusion of major multidisciplinary databases such as Scopus or Web of Science may limit coverage of important healthcare management and information systems literature. A short justification of this decision should be added to the limitations section.

 Third, the proposed process model is conceptually valuable, but its practical applicability could be enhanced by providing illustrative examples from the reviewed studies. Including concrete healthcare cases or organizational scenarios would make the framework more actionable and easier for readers to interpret.

 Finally, the manuscript would benefit from a more explicit discussion of ethical and governance considerations surrounding AI implementation in healthcare, such as data privacy, algorithmic bias, explainability, and workforce adaptation. These issues are increasingly central in AI-healthcare discussions and would strengthen the comprehensiveness of the review.

 Overall, the revised manuscript has improved considerably, and the author has addressed many of the previous concerns effectively. The study now presents a clearer theoretical contribution and a more coherent review structure. With minor additional refinements related to methodological detail, database justification, and contextual discussion, the manuscript would be suitable for indexing.

Are the rationale for, and objectives of, the Systematic Review clearly stated?

No

Is the statistical analysis and its interpretation appropriate?

Partly

If this is a Living Systematic Review, is the ‘living’ method appropriate and is the search schedule clearly defined and justified? (‘Living Systematic Review’ or a variation of this term should be included in the title.)

No

Are sufficient details of the methods and analysis provided to allow replication by others?

No

Are the conclusions drawn adequately supported by the results presented in the review?

No

Reviewer Expertise:

AI in healthcare

I confirm that I have read this submission and believe that I have an appropriate level of expertise to confirm that it is of an acceptable scientific standard.

# Reviewer report: Reviewer response for version 1

**DOI:** 10.5256/f1000research.174071.r382872

**Published:** 2025-05-19

**Authors:** Ameen A. Noor

**Affiliations:** 1 Mustansiriyah University, Baghdad, Baghdad Governorate, Iraq

1. The details of the analyses and methods are insufficient for replication by others.

 2. The research requires broader statistical analysis to clarify its application and whether it can be used in other areas.

 3. The review requires greater support to obtain more robust results.

 4. The methodology used is justified, but requires the use of more than one technique and its application to more than one dataset.

Are the rationale for, and objectives of, the Systematic Review clearly stated?

Yes

Is the statistical analysis and its interpretation appropriate?

Partly

If this is a Living Systematic Review, is the ‘living’ method appropriate and is the search schedule clearly defined and justified? (‘Living Systematic Review’ or a variation of this term should be included in the title.)

Partly

Are sufficient details of the methods and analysis provided to allow replication by others?

Partly

Are the conclusions drawn adequately supported by the results presented in the review?

Yes

Reviewer Expertise:

Multimedia, AI

I confirm that I have read this submission and believe that I have an appropriate level of expertise to confirm that it is of an acceptable scientific standard, however I have significant reservations, as outlined above.

# Response

**Published:** 2026-01-30

**Authors:** Dereje Ferede

**Affiliations:** College of Informatics, University of Gondar, Gondar, Amhara, Ethiopia

**Feedback 1: **The details of the analyses and methods are insufficient for replication by others.

**Responses: **Thank you for the feedback. I have substantially revised the entire methodology section, providing detailed explanations and literature-supported justifications to ensure that the study is transparent and reproducible. Please refer to the methodology section on pages 8–20, which comprehensively elaborates on the adopted approach, search strategy, data sources, analysis process, and PRISMA framework.

**Feedback 2: **The research requires broader statistical analysis to clarify its application and whether it can be used in other areas.

**Responses: **I have incorporated over 100 publications from 2015 to 2025 to clarify the application of the methodology. For further details, please refer to the methodology section on pages 8–20, which comprehensively elaborates on the approach I followed, the search strategy, data sources, analysis process, and PRISMA framework. Additionally, please refer to Appendix A, which provides detailed information on the reviewed articles.

**Feedback 3: **The review requires greater support to obtain more robust results.

**Responses: **I have refined the entire Results section by integrating findings from all 102 articles across the three databases, allowing for a more robust and reliable presentation of the results. Please refer to the Results section and Appendix A for detailed information on the reviewed articles.

**Feedback 4:  **The methodology used is justified, but requires the use of more than one technique and its application to more than one dataset.

**Responses: **I have made significant revisions to the entire Methodology section, providing detailed explanations and justifications supported by existing literature. Please refer to the Methodology section (pages 8–20), which thoroughly elaborates on the approach I followed, the search strategy, data sources, analysis process, and the PRISMA framework.

 I trust that these revisions adequately address your concerns.

 Thank you for your remarks, and I hope I have addressed all your concerns.

# Reviewer report: Reviewer response for version 1

**DOI:** 10.5256/f1000research.174071.r362024

**Published:** 2025-02-10

**Authors:** Ferry Fadzlul Rahman

**Affiliations:** 1 Universitas Muhammadiyah Kalimantan Timur, Samarinda, Indonesia

**"Artificial Intelligence (AI) and Healthcare Capabilities: A Systematic Literature Review"**, along with reasons that could justify its **rejection**:

1. **Limited Data Sources**
  - **Flaw:** The study relies exclusively on the PubMed database, ignoring other major academic sources such as IEEE Xplore, Scopus, and Web of Science.
  - **Reason for Rejection:** The exclusion of diverse sources limits the comprehensiveness and credibility of the findings, potentially leading to biased conclusions and an incomplete understanding of the topic.
2. **Weaknesses in Study Selection Criteria**
  - **Flaw:** The research includes only articles published after 2017 and written in English, excluding potentially valuable studies published earlier or in other languages. 
  - **Reason for Rejection:** This narrow selection criteria may lead to a significant omission of foundational research, reducing the study’s rigor and generalizability, particularly for regions where relevant studies might not be available in English.
3. **Lack of Justification for the Conceptual Framework**
  - **Flaw:** The proposed conceptual framework lacks a clear justification or comparison with existing models in the field.
  - **Reason for Rejection:** Without a robust theoretical foundation or validation, the framework's contribution to the academic field is questionable, making it difficult to assess its novelty and practical relevance.
4. **Insufficient Contextual Analysis of the Global South**
  - **Flaw:** Despite emphasizing the importance of AI adoption in developing countries, the study does not provide an in-depth discussion of contextual challenges such as technological infrastructure, regulatory policies, and readiness for AI adoption.
  - **Reason for Rejection:** The lack of context-specific insights weakens the study's practical applicability and relevance for stakeholders in the Global South, contradicting the study’s stated focus.
5. **Inadequate Discussion of Ethical and Privacy Issues**
  - **Flaw:** The study fails to comprehensively address crucial ethical and privacy concerns associated with AI implementation in healthcare.
  - **Reason for Rejection:** Ethical and privacy considerations are fundamental in healthcare AI applications; neglecting these aspects undermines the study's completeness and practical impact.
6. **Methodological Limitations**
  - **Flaw:** While the study follows the PRISMA framework, it lacks a thorough explanation of how validity and reliability were ensured in the study selection and screening processes.
  - **Reason for Rejection:** Weak methodological rigor can lead to inconsistent or unreliable findings, making the study unsuitable for indexing in a high-impact journal.
7. **Lack of Clear Practical Implications**
  - **Flaw:** The study claims to provide practical insights for healthcare practitioners and policymakers, but the recommendations are too general and lack actionable strategies.
  - **Reason for Rejection:** Without clear, evidence-based recommendations, the study offers limited value to practitioners and decision-makers, diminishing its practical significance.

Are the rationale for, and objectives of, the Systematic Review clearly stated?

No

Is the statistical analysis and its interpretation appropriate?

Partly

If this is a Living Systematic Review, is the ‘living’ method appropriate and is the search schedule clearly defined and justified? (‘Living Systematic Review’ or a variation of this term should be included in the title.)

No

Are sufficient details of the methods and analysis provided to allow replication by others?

No

Are the conclusions drawn adequately supported by the results presented in the review?

No

Reviewer Expertise:

AI in healthcare

I confirm that I have read this submission and believe that I have an appropriate level of expertise to state that I do not consider it to be of an acceptable scientific standard, for reasons outlined above.

# Response

**Published:** 2026-01-30

**Authors:** Dereje Ferede

**Affiliations:** College of Informatics, University of Gondar, Gondar, Amhara, Ethiopia

**Feedback 1: **Limited Data Sources: Flaw: The study relies exclusively on the PubMed database, ignoring other major academic sources such as IEEE Xplore, Scopus, and Web of Science. Reason for Rejection: The exclusion of diverse sources limits the comprehensiveness and credibility of the findings, potentially leading to biased conclusions and an incomplete understanding of the topic.

**Responses: **To address the limitation of available data sources, I conducted the literature search using PubMed, IEEE Xplore, and the AIS eLibrary. These databases were selected because they are either open access or accessible through my institutional subscription. Other databases were excluded due to lack of access, which is acknowledged as a limitation of this study. As stated on page 8, subsection 3.2 (Data Sources and Search Strategy), a systematic literature search was conducted using databases that are either open access or accessible through institutional subscriptions. PubMed and IEEE Xplore were used as open-access databases, while the AIS eLibrary was accessed via institutional subscription. These databases were selected for their strong focus on health sciences and technology, ensuring comprehensive coverage of research on AI in healthcare. Additionally, as detailed in Section 3.3 and summarized in Table 2 (Inclusion and Exclusion Criteria), publications from 2015 to 2025 were included in the review. For further details, please refer to the Methodology section.

**Feedback 2:** Weaknesses in Study Selection Criteria. Flaw: The research includes only articles published after 2017 and written in English, excluding potentially valuable studies published earlier or in other languages.  Reason for Rejection: This narrow selection criteria may lead to a significant omission of foundational research, reducing the study’s rigor and generalizability, particularly for regions where relevant studies might not be available in English.

**Responses: **I expanded the study selection criteria beyond the initial three databases. Specifically, I extended the publication period from 2017–2024 to 2015–2025 and limited inclusion to studies published in English. Studies published in other languages were excluded due to my lack of proficiency in those languages; this constraint was explicitly acknowledged as a limitation of the study. For instance, on page 8, Subsection 3.2 (Data Sources and Search Strategy), I stated the following: a systematic literature search was conducted using databases that are either open-access or accessible through my institution. PubMed and IEEE Xplore were used as open-access databases, while the AIS eLibrary was accessed via institutional subscription. These databases were selected for their strong coverage of health sciences and technology, ensuring comprehensive retrieval of literature on AI in healthcare. In addition, publications from 2015 to 2025 were included, as specified in Section 3.3, particularly in Table 2 (Inclusion and Exclusion Criteria).

**Feedback 3:** Lack of Justification for the Conceptual Framework. Flaw: The proposed conceptual framework lacks a clear justification or comparison with existing models in the field. Reason for Rejection: Without a robust theoretical foundation or validation, the framework's contribution to the academic field is questionable, making it difficult to assess its novelty and practical relevance.

**Responses: **I refined the framework into a complete process model, incorporating theoretically grounded propositions supported by justifications from the literature. For example, on page 32 in the Discussion section, Figure 3 presents a substantially revised and entirely new version of the framework, directly addressing your concern. As stated in the manuscript: In addressing the research question—how healthcare organizations build and leverage AI-enabled capabilities to create organizational value—this study proposes a process model (Figure 3) that conceptualizes AI-enabled value creation as the outcome of a co-evolutionary process between AI tools and organizational micro-foundations. Rather than resulting from the mere adoption of AI technologies, value creation emerges through the dynamic interplay among technology, human actors, and organizational processes. Grounded in dynamic capability theory, the findings demonstrate how AI enables healthcare organizations to sense opportunities and threats, seize value-creating initiatives, and transform existing routines in response to environmental uncertainty, while simultaneously navigating the associated challenges.

**Feedback 4: **Insufficient Contextual Analysis of the Global South. Flaw: Despite emphasizing the importance of AI adoption in developing countries, the study does not provide an in-depth discussion of contextual challenges such as technological infrastructure, regulatory policies, and readiness for AI adoption. Reason for Rejection: The lack of context-specific insights weakens the study's practical applicability and relevance for stakeholders in the Global South, contradicting the study’s stated focus.

**Responses: **I revised the manuscript to focus on healthcare organizations in general, removing references to the “Global South” and “Global North.” Within this broader scope, I discussed the challenges faced by healthcare organizations as identified through the literature review. In addition, I elaborated on both the practical and theoretical implications of the study in the Discussion section. Please refer to Appendix A for the list of the 102 reviewed studies, and to the Discussion section for a detailed presentation of the study’s implications for both theory and practice.

**Feedback 5: **Inadequate Discussion of Ethical and Privacy Issues. Flaw: The study fails to comprehensively address crucial ethical and privacy concerns associated with AI implementation in healthcare. Reason for Rejection: Ethical and privacy considerations are fundamental in healthcare AI applications; neglecting these aspects undermines the study's completeness and practical impact.

**Responses: **I incorporated ethical considerations and other challenges associated with AI adoption in healthcare organizations into both the literature review and the process model, emphasizing how these issues shape and constrain healthcare organizational capabilities. For example, the process model includes “challenges in AI-enabled healthcare” as a core dimension. This dimension comprises three key elements: (1) trust, transparency, and professional resistance; (2) technical and data-related limitations; and (3) ethical, legal, and regulatory frictions affecting healthcare organizations. These challenges are discussed in both the Results and Discussion sections. I hope this adequately addresses your concern.

**Feedback 6: **Methodological Limitations. Flaw: While the study follows the PRISMA framework, it lacks a thorough explanation of how validity and reliability were ensured in the study selection and screening processes. Reason for Rejection: Weak methodological rigor can lead to inconsistent or unreliable findings, making the study unsuitable for indexing in a high-impact journal.

**Responses: **I completely revised the methodology section, providing a detailed explanation of how and why the PRISMA framework was applied in this study. I literature. Please refer to the entire Methodology section (pages 8–20), which comprehensively elaborates the research approach, search strategy, data sources, analysis process, and the application of the also clarified the procedures used to ensure validity and reliability during the study selection and screening process, supported by relevant methodological PRISMA framework.

**Feedback 7: **Lack of Clear Practical Implications. Flaw: The study claims to provide practical insights for healthcare practitioners and policymakers, but the recommendations are too general and lack actionable strategies. Reason for Rejection: Without clear, evidence-based recommendations, the study offers limited value to practitioners and decision-makers, diminishing its practical significance.

**Responses: **I refined the practical implications for key stakeholders—including healthcare organizations, clinicians, policymakers, and AI developers—by providing clear and well-justified implications for each group. For example, in Section 5.1 (Implications for Theory and Practice), I explicitly outline four categories of practical implications: (1) implications for policymakers and regulators; (2) implications for AI developers and vendors; (3) implications for clinicians and healthcare professionals; and (4) implications for healthcare organizations.

 I trust that these revisions adequately address your concerns.
